# Locus of Adhesion and Autoaggregation (LAA), a pathogenicity island present in emerging Shiga Toxin–producing *Escherichia coli* strains

**DOI:** 10.1038/s41598-017-06999-y

**Published:** 2017-08-01

**Authors:** David A. Montero, Juliana Velasco, Felipe Del Canto, Jose L. Puente, Nora L. Padola, David A. Rasko, Mauricio Farfán, Juan C. Salazar, Roberto Vidal

**Affiliations:** 1 Programa de Microbiología y Micología, Instituto de Ciencias Biomédicas, Facultad de Medicina, Universidad de Chile, Santiago, Chile; 2 Servicio de Urgencia Infantil, Hospital Clínico de la Universidad de Chile “Dr. José Joaquín Aguirre”, Santiago, Chile; 3 Departamento de Microbiología Molecular, Instituto de Biotecnología, Universidad Nacional Autónoma de México, Cuernavaca, Mexico; 4 Centro de Investigación Veterinaria Tandil, CONICET-CIC, Facultad de Ciencias Veterinarias, UNCPBA, Tandil, Argentina; 5 Department of Microbiology and Immunology, University of Maryland School of Medicine, Baltimore, Maryland USA; 6 Centro de Estudios Moleculares, Departamento de Pediatría, Hospital Dr. Luis Calvo Mackenna, Facultad de Medicina, Universidad de Chile, Santiago, Chile; 7 Instituto Milenio de Inmunología e Inmunoterapia, Facultad de Medicina, Universidad de Chile, Santiago, Chile

## Abstract

Shiga Toxin-producing *Escherichia coli* (STEC) are a group of foodborne pathogens associated with diarrhea, dysentery, hemorrhagic colitis (HC) and hemolytic uremic syndrome (HUS). Shiga toxins are the major virulence factor of these pathogens, however adhesion and colonization to the human intestine is required for STEC pathogenesis. A subset of STEC strains carry the Locus of Enterocyte Effacement (LEE) pathogenicity island (PAI), which encodes genes that mediate the colonization of the human intestine. While LEE-positive STEC strains have traditionally been associated with human disease, the burden of disease caused by STEC strains that lacks LEE (LEE-negative) has increased recently in several countries; however, in the absence of LEE, the molecular pathogenic mechanisms by STEC strains are unknown. Here we report a 86-kb mosaic PAI composed of four modules that encode 80 genes, including novel and known virulence factors associated with adherence and autoaggregation. Therefore, we named this PAI as Locus of Adhesion and Autoaggregation (LAA). Phylogenomic analysis using whole-genome sequences of STEC strains available in the NCBI database indicates that LAA PAI is exclusively present in a subset of emerging LEE-negative STEC strains, including strains isolated from HC and HUS cases. We suggest that the acquisition of this PAI is a recent evolutionary event, which may contribute to the emergence of these STEC.

## Introduction

Shiga Toxin-producing *Escherichia coli* (STEC) are a group of foodborne pathogens associated with gastrointestinal diseases, including acute diarrhea and dysentery. Annually, STEC causes over two million cases of acute illness worldwide^1^. Importantly, STEC infection may progress to severe diseases such as hemorrhagic colitis (HC) and hemolytic uremic syndrome (HUS)^2^. In humans, STEC pathogenesis involves the initial adhesion of the bacteria to the intestinal epithelium in the ileum, later colonization of the colon and production of Shiga toxin (Stx), thereby impairing epithelial barrier function and ion transport, causing diarrhea^3, 4^. Stx may reach the bloodstream and disseminate to extra-intestinal tissues, producing more severe diseases such as HUS that may result in death. Thus, although Stx is considered *sine qua non* of virulence, adhesion to the intestinal mucosa is a required first step for STEC pathogenesis^5^. In particular, a subset of STEC strains use an adhesion mechanism called attaching-and-effacing (A/E) lesion, which is characterized by the alteration of the architecture and physiology of the colon epithelial cells. This pathogenic process is mediated by genes encoded in the **L**ocus of **E**nterocyte **E**ffacement (LEE) pathogenicity island (PAI)^6^. To date, STEC strains that are LEE-positive, such as O157:H7 and several serotypes belonging to the “big six” non-O157 STEC O serogroups (O26, O45, O103, O111, O121 and O145), have been the most frequently associated with outbreaks and/or severe illness^7^. Consequently, in the context of STEC infection, the presence of LEE is considered a risk factor for the development of HUS^8^.

However, STEC strains that do not carry LEE (LEE-negative) have also been isolated from cases of severe illness^5^. Indeed, there has been an increase in the number of reports of clinical LEE-negative STEC strains that belong to serogroups O91, O113 and O174^9–11^. For instance, there has been an increase in the detection of O91 strains in Germany from ~5% of all STEC strains isolated from humans in 1999 to ~15% in 2012 and 2013^12^. Similarly from 2007 to 2012, the serogroups O91 and O113 were among the most common non-O157 serogroups associated with human disease in Netherlands^13^. Recently, the serogroup O174 was identified as one of the four non-O157 serogroups most commonly associated with HUS in Argentina^14^. Nevertheless, in the absence of LEE, the molecular mechanisms by which these strains adhere to the host intestinal epithelium remain largely unknown^15^.

In light of these observations, our group is currently investigating the emergence of LEE-negative STEC strains of clinical relevance. In a previous report, we identified a member of the Heat-resistant agglutinin family (Hra Family) produced by the LEE-negative O113:H21 STEC strain E045-00, which is seroreactive to sera from patients with HUS^16^. In this study, we characterized this antigen named **He**magglutinin from **S**higa toxin-producing *E. coli* (Hes). Much like other members of the Hra family, Hes is a virulence factor that participates in several colonization-associated phenotypes, including hemagglutination, adhesion and autoaggregation. More importantly, we show that the *hes* gene is localized in a 86-kb mosaic PAI composed of 80 genes organized into four modules, one of them (module III) previously described by Shen *et al*.^17^. Here we report the complete sequence of this PAI, in which other virulence factors participating in adhesion and autoaggregation are also encoded, such as Iha^18^ and Ag43^19^, respectively. Consequently, this PAI was named the **L**ocus of **A**dhesion and **A**utoaggregation (LAA). We also determined the distribution of this PAI among STEC strains isolated from different sources, showing its presence in a subset of LEE-negative STEC strains, some of which were isolated from cases of HC or HUS. Remarkably, our analyses suggest the acquisition of LAA is probably a recent evolutionary event, which may contribute to the emergence of these pathogens. Thus, this study is a step forward toward an understanding the evolution, emergence and pathogenicity of this subset of STEC strains. Additionally, the identification of this PAI will be useful in epidemiological studies that assess the public health risk of STEC.

## Results

### The Hra Family includes a novel multifunctional protein that is widely distributed in LEE-negative STEC strains

The Hra family is composed of the integral outer membrane proteins Hra1, Hra2, Tia and Hek, which share considerable amino acid sequence similarity but participate in different colonization-associated phenotypes^20^. Hra1 participates in hemagglutination, autoaggregation, biofilm formation and aggregative adherence^21^; Hra2 participates in adhesion^20^; Tia in adhesion and invasion^22^; and Hek in hemagglutination, autoaggregation, adhesion and invasion^23^. In a previous study, we identified a member of the Hra family in the outer membrane protein extract of the LEE-negative O113:H21 STEC strain E045‐00^16^. The analysis of this protein by mass spectrometry (MALDI TOF/TOF) suggested that it was the Hek protein. During subsequent analysis aimed at determining whether Hek was present in other STEC strains, the amino acid sequence of this protein was used to perform BLASTp searches against the NCBI nr database. Nevertheless, the Hek protein was not detected in any STEC strains. In contrast, we found an allelic variant (GenBank accession: EGW68377) of the Hra family in the LEE-negative O91:H21 STEC strain B2F1, which has 90%, 65%, 65% and 86% amino acid identity with the Hra1, Hra2, Tia and Hek proteins, respectively, and that primarily exhibits amino acid substitutions in predicted loops exposed to the extracellular space (Supplementary Fig. 1). This variant has not been described previously. Therefore, following the nomenclature used for members of the Hra family, we named it **He**magglutinin from **S**higa toxin-producing *E. coli* (Hes).

To determine the distribution of *hes* in our culture collection (167 STEC strains, including 48 LEE-negative STEC, and 12 fecal *E. coli* isolated from healthy subjects), PCR analysis was performed using primers *hes*_*det1* + *hes*_*det2* (Supplementary Table 1), which are specific for this gene and do not amplify other allelic variants of the Hra family (Supplementary Note 1). Strains positive for *hes* were then analyzed by PCR using the primers *hes*_*for* + *hes*_*rev*, which amplify the complete nucleotide sequence of *hes* and other members of the Hra family, and the PCR products obtained were sequenced. Notably, 60% (29/48) of the LEE-negative STEC strains were positive for *hes*, including isolates of serotypes O91:H21, O113:H21 and O174:H21 (Supplementary Table 2). Nevertheless, *hes* was not detected in two strains of the serotype O113:H21. Importantly, the E045-00 strain was positive for *hes*, indicating that the peptide originally mis-identified as Hek is indeed Hes. In addition, sequencing of PCR products indicated that *hes* sequence is 99.7% conserved (data not shown). In contrast, this gene was not detected in any of the LEE-positive STEC strains (0/119) or fecal isolates (0/12). These results indicate that Hes is a novel variant of the Hra family that is widely distributed in LEE-negative STEC strains.

**Figure 1:**
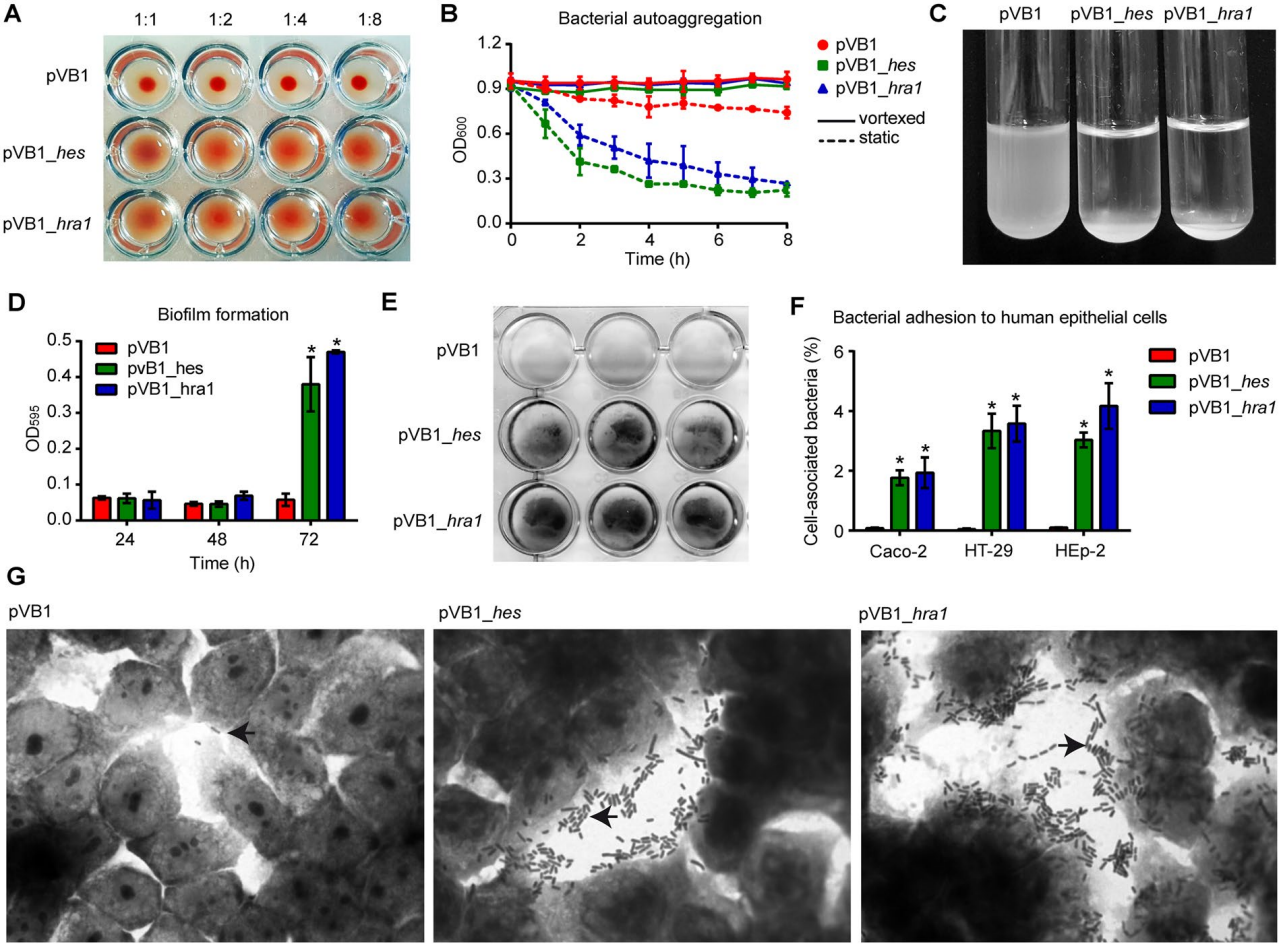
Functional characterization of the Hes protein. *E. coli* HB101 strain transformed with pVB1, pVB1_*hes* or pVB1_*hra1* plasmids was evaluated in its capacity of agglutination sheep erythrocytes, bacterial autoaggregation, biofilm formation and adhesion to human epithelial cells. The HB101/pVB1 and HB101/pVB1-*hra1* were negative and positive controls in all assays, respectively. (**A**) Hemagglutination assay. Bacteria were assessed in 1:1, 1:2, 1:4 and 1:8 dilutions in PBS. A positive result is indicated by formation of a red film, and a negative result is indicated by a red sediment at the bottom of the well. (**B**) Bacterial autoaggregation. Overnight cultures were centrifuged and re-suspended in PBS to an optical density of ~0.9 measured at 600 nm (OD_600_). Bacterial suspensions were left static for 8 h. The OD_600_ of suspension withdrawn from 1 cm from the surface was measured (dashed line). For each strain, a parallel tube (continuous line) was vortexed before measurements were taken. Autoaggregation ability is proportionate to the distance between the measurement plots of vortexed and static suspension. Error bars represent standard deviation (s.d.) (n = 2). (**C**) Bacterial suspension after static incubation at room temperature for 8 h. (**D**) Biofilm formation measured at 24, 48 and 72 h by crystal violet staining and elution. Biofilms were quantified as OD_595_ of eluted crystal violet. Error bars represent s.d. (n = 3). (**E**) Biofilm produced after 72 h on polystyrene surface. (**F**) Bacterial adhesion to human epithelial cells (Caco-2, HT-29 and Hep-2 cells). Data are expressed as the percentage of the initial inoculum recovered after 30 min of infection at a multiplicity of infection of 10 bacteria per cell. Error bars represent s.d. (n = 3). (**G**) Giemsa staining of adherent bacteria on HT-29 cell monolayers visualized by light microscopy. Magnification, ×1,000. Arrows indicates adherent bacteria. *P < 0.005 by Student’s t-test (two-tailed) relative to the HB101/pVB1 strain.

Given the biological role of members of the Hra family, we evaluated whether the *hes* gene product is capable of conferring colonization-associated phenotypes to the non-adherent *E. coli* HB101 strain. When expressed under the control of an inducible promoter, *hes* confers the capacity of agglutination of sheep erythrocytes (Fig. 1A) and autoaggregation (Fig. 1B,C) to the HB101 strain. It also promotes biofilm formation at 72 h (Fig. 1D,E). *In vitro* adhesion and invasion assays indicate that *hes* significantly increases the adherence of this strain to epithelial cells (Fig. 1F), in an aggregative pattern (Fig. 1G), but does not promote invasion of epithelial cells (not shown). These results indicate that Hes is functional and confers *E. coli* with phenotypic characteristics typical of other members of the Hra family.

### *hes* is encoded in a pathogenicity island

**Figure 2:**
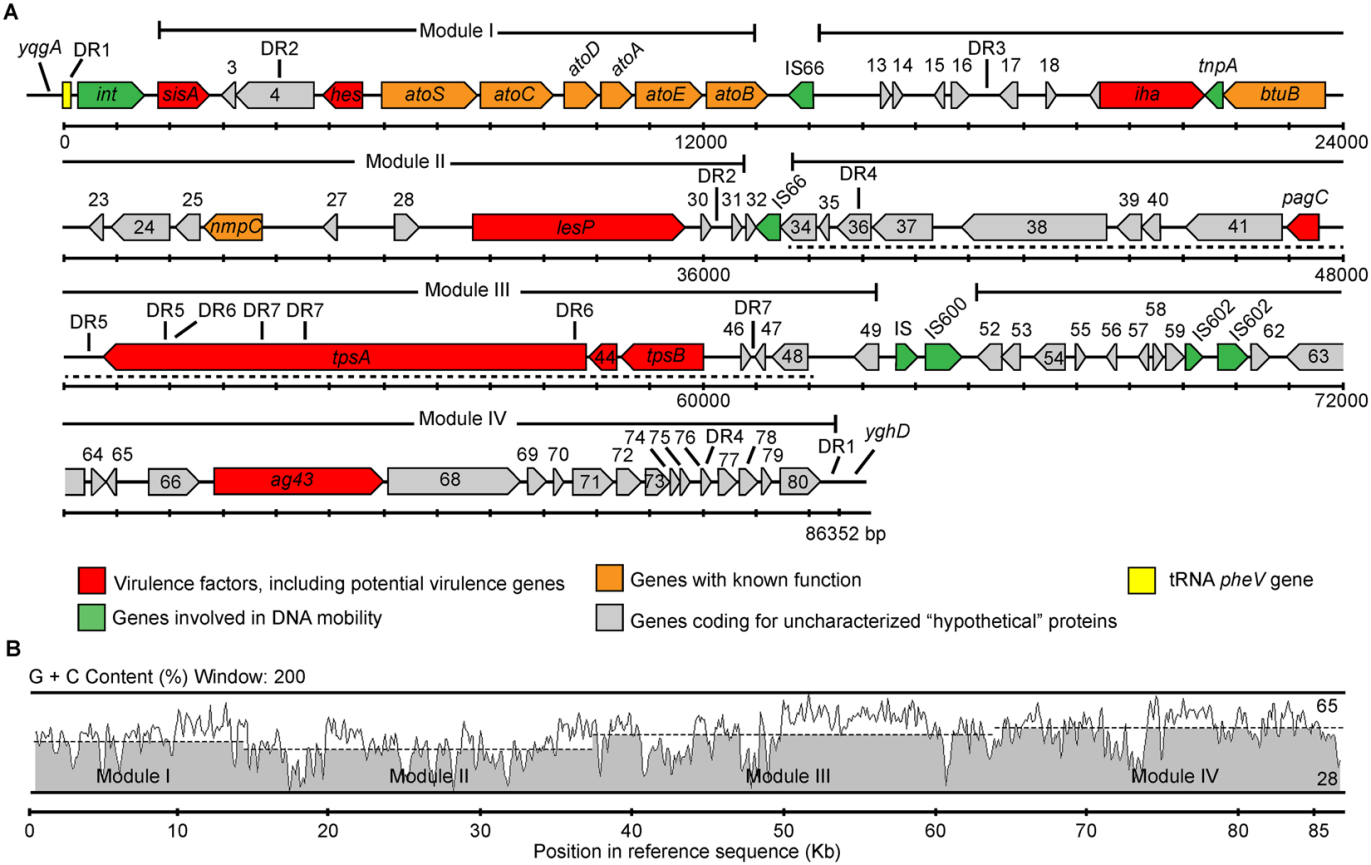
Genetic structure of the Locus of Adherence and Autoaggregation (LAA) pathogenicity island. (**A**) Predicted genes and direction of transcription are represented as block arrows. Open reading frames (ORFs) are color coded according to gene function, as indicated by legend at the bottom. Names of some genes are shown. Features of each ORF are listed in Supplementary Table 3. This DNA region is located between positions 385,984–472,336 bp in the contig #25 (GenBank accession: AFDQ01000026) of the draft genome of the B2F1 strain^56^. This pathogenicity island has a mosaic structure that is organized in modules flanked by IS elements and/or DR sequences. Dotted line, DNA region reported by Shen *et al*.^17^. (**B**) G + C content of each module: I (48.6%), II (43.8%), III (50.2%) and IV (51.7%).

Some members of the Hra family, such as Hek and Tia, are encoded in PAIs^24, 25^. In fact, one of these PAIs, the Subtilase-Encoding Pathogenicity Island, has been identified in LEE-negative STEC strains isolated from humans and animals^25^. Given these reports and the mutually exclusive distribution of *hes* with LEE in STEC strains, we sought to determine whether this gene is harbored in a mobile genetic element. *In silico* analysis of the boundaries of the *hes* gene in the draft genome of STEC strain B2F1 (see methods for details), showed that an integrase-encoding gene and the *pheV* tRNA gene are located 3,342 and 4,805 bp upstream of the *hes* stop codon, respectively (Fig. 2A). Also, 23-bp imperfect direct repeats (DR1) that corresponds to the 3′ end of the *pheV* gene were identified 80,626 bp downstream of the *hes* start codon. These DR1 sequences flank a 86,256-bp DNA region with a G + C content of 48%, which is less than the 51% G + C exhibited by the B2F1 chromosome. Additionally, other direct repeats (DR2 to DR7) and several insertion sequence (IS) elements were identified within this chromosomal region. The *yqgA* and *yghD* genes are located next to the DR1 sequences, with the first located upstream and the second downstream. Genome analysis of the laboratory strain *E. coli* K-12 MG1655 indicated that both genes are located in the vicinity of the *pheV* gene (not shown). Thus, *hes* is encoded in a 86,256-bp DNA region that is inserted in the *pheV* gene in the B2F1 strain. In this DNA region, 80 ORFs (open reading frames) were identified, including genes with known functions, some of which are associated with pathogenicity in STEC, as well as several genes and pseudogenes that code for hypothetical proteins (*vide infra*) (Fig. 2A and Supplementary Table 3). Among the genes coding for virulence factors identified, in addition to *hes* (ORF5), there are other genes that code for adhesins such as Iha (ORF20)^18^ and Ag43 (ORF67), which also promotes autoaggregation^19^. Given that this cluster of genes is related to adhesion and autoaggregation, we have named this DNA region as **L**ocus of **A**dhesion and **A**utoaggregation (LAA). Collectively, these results show that LAA exhibits several features typical of a PAI.

### LAA encodes several known and hypothetical proteins, including virulence factors other than adhesins

In addition to *hes*, LAA carries genes encoding functions other than adhesion and autoaggregation (Supplementary Table 3). The *nmpC* gene (ORF26) has been associated with heat resistance in *E. coli* ^26^, while the NmpC protein of STEC strain E045-00 has been shown to be seroreactive only with HUS sera^16^. The *sisA* gene (ORF2) product has the ability to attenuate the host inflammatory response induced by uropathogenic *E. coli* strains^27^. Four additional ORFs encoding potential virulence factors are: ORF29, which encodes for a novel variant of a Serine Protease Autotransporter of Enterobacteriaceae (SPATE), herein called the **L**AA **e**ncoded **SP**ATE (LesP) (Supplementary Table 4); ORF42, which encodes a protein sharing 60.6% similarity with the *Salmonella enterica* PagC protein involved in serum resistance phenotype^28^; and ORF43 and ORF45 (*tpsA* and *tpsB* genes, respectively), which encode a two-partner secretion system that in Gram-negative bacteria participates in different virulence phenotypes^29^. However, the biological function of these hypothetical proteins remains uncharacterized.

Several transcriptional regulators are also encoded in PAIs^30^. These proteins may control the expression of genes located at PAIs and/or elsewhere in the genome. Two major classes of these transcriptional regulators are proteins of the AraC family and two-component signal transduction systems. ORF28 encodes a hypothetical protein of the AraC family that is also present in the genomic island called the **L**ocus of **P**roteolysis **A**ctivity (LPA)^31^. ORF6 and ORF7 encode the AtoS-AtoC two-component system that positively regulates the expression of the *atoDAEB* operon (ORF8–ORF11)^32^. The potential involvement of these regulators in the expression of LAA-encoded functions and virulence is a matter of current investigation.

### LAA is a pathogenicity island that is present in LEE-negative STEC strains

**Figure 3:**
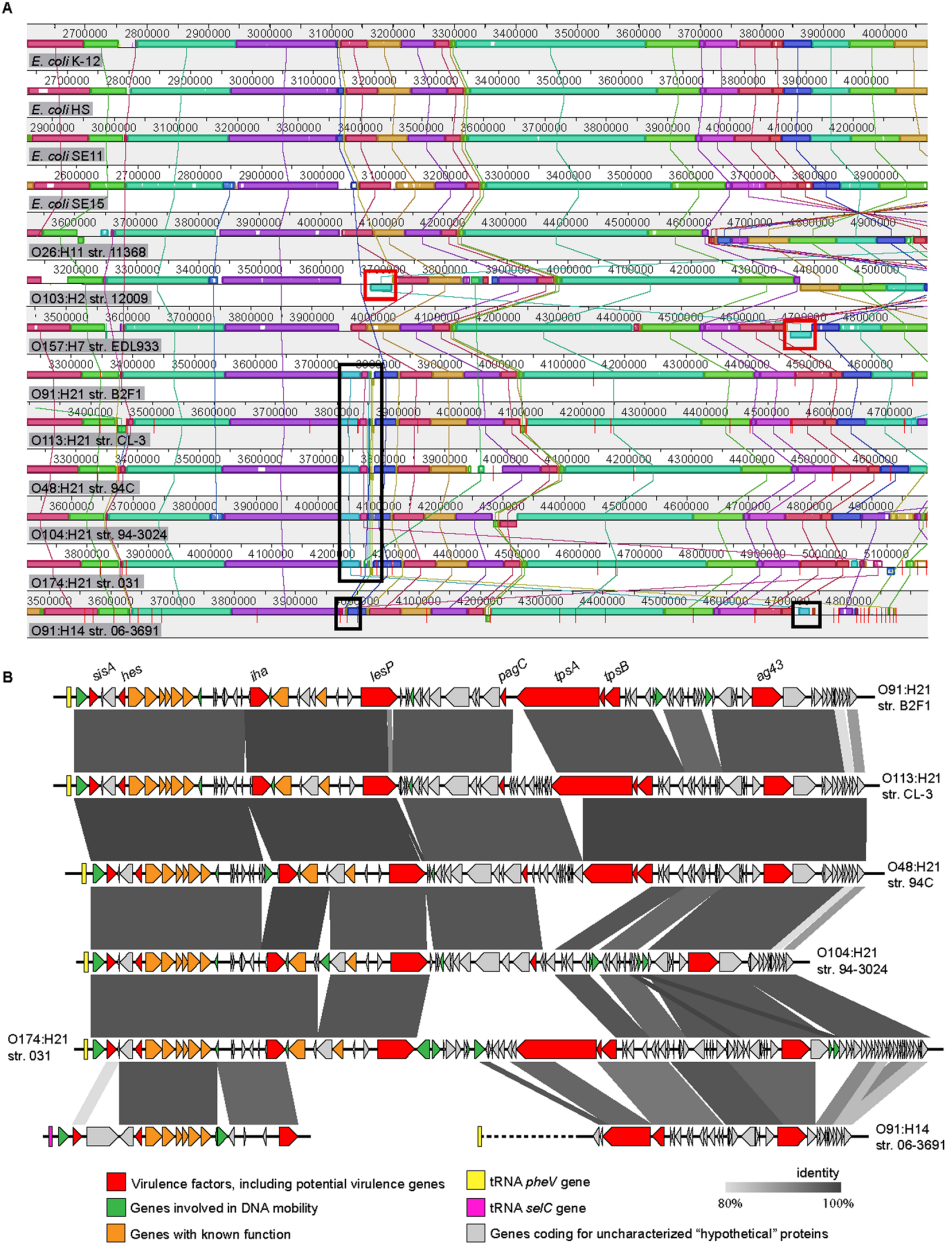
Identification of the LAA pathogenicity island in LEE-negative STEC strains. (**A**) Alignment between genomes of representative LEE-negative STEC strains (O48:H21, O91:H14, O91:H21, O104:H21, O113:H21 and O174:H21), LEE-positive STEC strains (O26:H21, O103:H2 and O157:H7) and the genomes of *E. coli* K-12 MG1655 and commensal *E. coli* strains (HS, SE11 and SE15). Alignment was performed using progressiveMauve^33^. Genome sequences used are listed in Table S5. The complete genome sequence of *E. coli* K-12 substr. MG1655 was used as the reference sequence. Colored blocks represent collinear and homologous regions. Non-colored areas represent unaligned sequences that may be genome-specific. Inverted regions are identified by boxes below the central line. Regions between consecutive red lines indicate individual contigs. The location of the LAA pathogenicity island is show by black rectangles. The location of the LEE pathogenicity island is shown by red rectangles. (**B**) A comparison of the genetic structure of the LAA pathogenicity island present in six LEE-negative STEC strains. Predicted genes and the direction of transcription are represented as block arrows. Open reading frames (ORFs) are color coded according to gene function, as indicated in the legend at the bottom. Names of virulence genes are show. Conserved regions are shaded in grey and the intensity of the color indicates nucleotide identity levels, from 80 to 100%. The figure was prepared using EasyFig^53^.

Since LAA exhibits several features of a PAI, we hypothesized that this locus should also be present in other STEC strains, but not in commensal strains. To test this, we performed alignments between representative genomes (draft or complete) of LEE-positive and LEE-negative STEC and commensal strains using progressiveMauve^33^ (Fig. 3). As for strain B2F1, LAA was identified downstream of the *pheV* gene in the LEE-negative STEC strains, with exception of O91:H14 str. 06-3691 where a region only harboring genes ORF2 to ORF20 (18.5-kb) was located next to the *selC* tRNA gene, and a second region containing genes ORF43 (truncated) to ORF80 was located next to the *pheV*, while genes ORF21 to ORF40 were not present. In contrast, LAA was not identified in K12, commensal or LEE-positive STEC strains. Thus, these results suggest that LAA is uniquely associated with LEE-negative STEC strains.

### LAA has a mosaic structure organized into four modules

The presence of LAA fragments at locations other than the *pheV* locus, along with the identification of several DR sequences and IS elements, raises the question as to whether this locus has a mosaic structure. Initially, we analyzed its nucleotide sequence searching for DNA regions flanked by IS elements, DR sequences and/or having different G + C content, which may correspond to modules. Four fragments (modules) with these features were identified (Fig. 2A): module I (13-kb) from the *pheV* gene to ORF11 is flanked at the 3′ end by a putative transposase of the IS66 family (ORF12)﻿; module II (23-kb) from ORF13 to ORF32 is flanked at the 3′ end by a putative transposase of the IS66 family (ORF33); module III (26-kb) from ORF34 to ORF49 is flanked at the 3′ end by genes encoding putative transposases of the of the IS600 family (ORF50 and ORF51) ; and module IV (21-kb) from ORF52 to ORF80 located at the 3′ end of LAA. Additionally, all of these DNA regions have different G + C content (Fig. 2B), supporting the idea that they potentially have different genetic origins.

**Figure 4:**
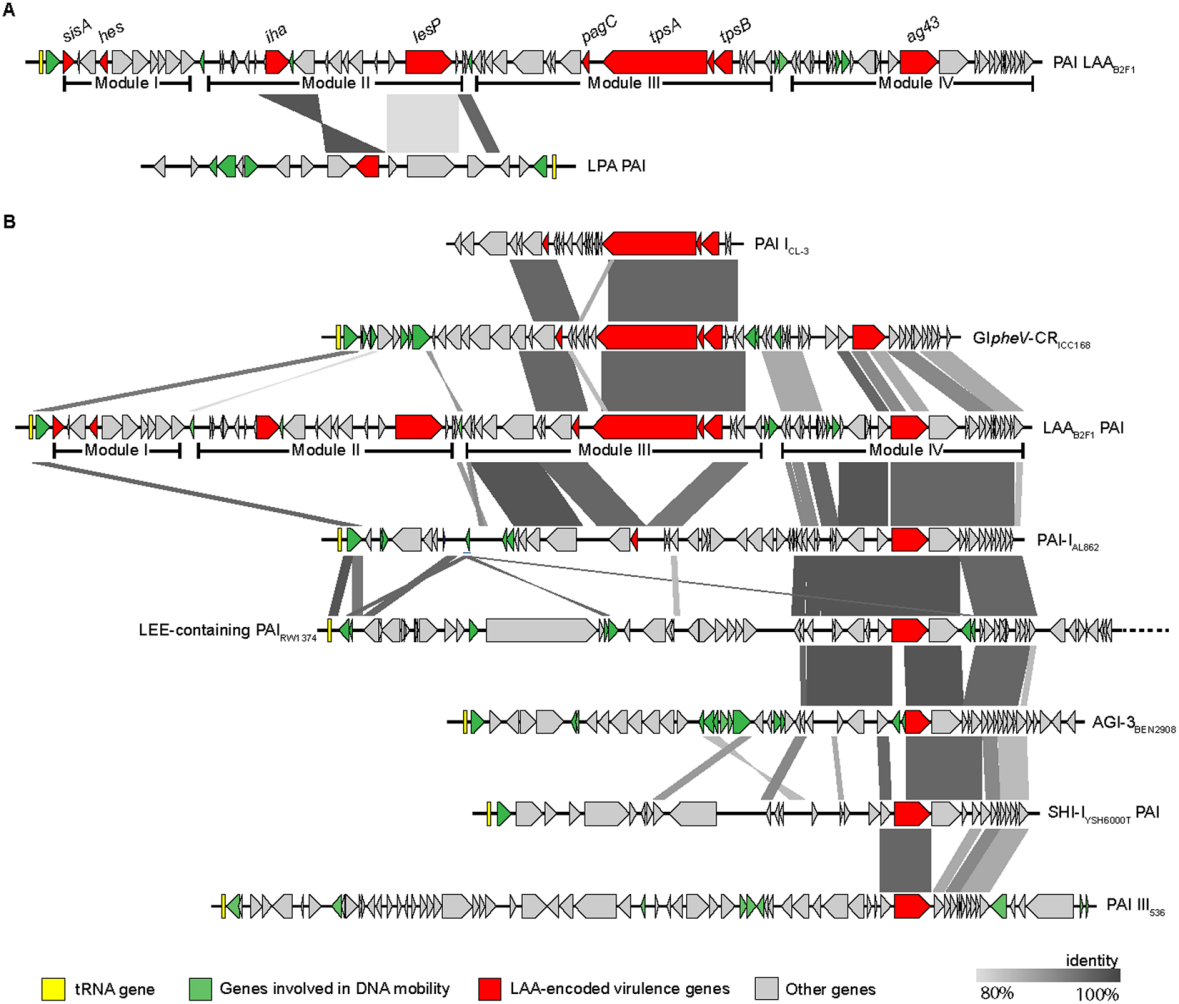
Comparison of the genetic structure of LAA_B2F1_ and related pathogenicity islands. Predicted genes and the direction of transcription are represented as block arrows. Open reading fames (ORFs) are color coded according to the legend at the bottom. The names of LAA-encoded virulence genes are indicated in the upper panel. Conserved regions are shaded in grey and the intensity of the color indicates nucleotide identity levels, from 80 to 100%. (**A**) Comparison between module II of LAA and the LPA pathogenicity island (PAI) (accession number: AJ278144). (**B**) Comparison between module III and IV of LAA and the PAI-I_CL3_ (accession number: AY275838), GIpheV-CRICC168, PAI-I_AL862_ (accession number: GQ497943), LEE-containing PAI_RW1374_ (accession number: AJ303141; note that this PAI is only partially shown), PAI AGI-3_BEN2908_ (accession number: AY857617), PAI SHI-I_YSH6000T_ (accession number: AF200692) and PAI-III_536_ (accession number: X16664). The figure was prepared using EasyFig^53^.

Next, we searched for these modules in the Pathogenicity Island Database v 2.0^34^ and found a number of other PAIs with DNA regions that shared more than 80% identity with them, with the exception of module I (Fig. 4). Module III was previously described in the LEE-negative STEC O113:H21 str. CL3 and called PAI I_CL3_ (Fig. 2A, dotted line)^17^. A similar gene cluster is found in the genomic island GI*pheV*-CR_ICC168_ of *C. rodentium* ^35^ and as part of the PAI-I_AL862_ with the deletion of the two-partner secretion system (ORF43-ORF45) (Fig. 4). On the other hand, module II shares homology with the LPA PAI. Likewise, module IV resembles a cluster of genes present in several PAIs (Fig. 4B). Collectively, these data confirm the mosaic structure of LAA and the diverse distribution of its modules.

### LAA may be present as a “complete” (with all four modules) or an “incomplete” (<4 modules) structure. The complete LAA structure is present in strains that cause severe diseases

At this point, it was interesting to investigate the distribution of LAA modules among *E. coli* strains. Accordingly, we developed a multiplex PCR assay for simultaneous detection of modules I, II and III. It was not possible to design specific primers for module IV, as its genes are widely distributed in *E. coli*. Pairs of primers LAA1_for + LAA1_rev and LAA2_for + LAA2_rev were designed to amplify modules I and II, respectively. A third pair of primers (ms3_for + ms3_rev), reported by Girardeau *et al*.^35^, was used for the amplification of module III (Supplementary Table 1 and Supplementary Fig. 2). With these primers, we examined the presence of the first three modules in our culture collection. As expected, PCR products were obtained only in LEE-negative STEC strains. Among them, 24/48 strains (50%) were positive for all three modules and 6/48 strains (12.5%) for two modules (Supplementary Table 2). These results demonstrate that LAA is associated with LEE-negative STEC strains and suggest that its modules might mobilize (be acquired or lost) independently of the complete structure.

**Figure 5:**
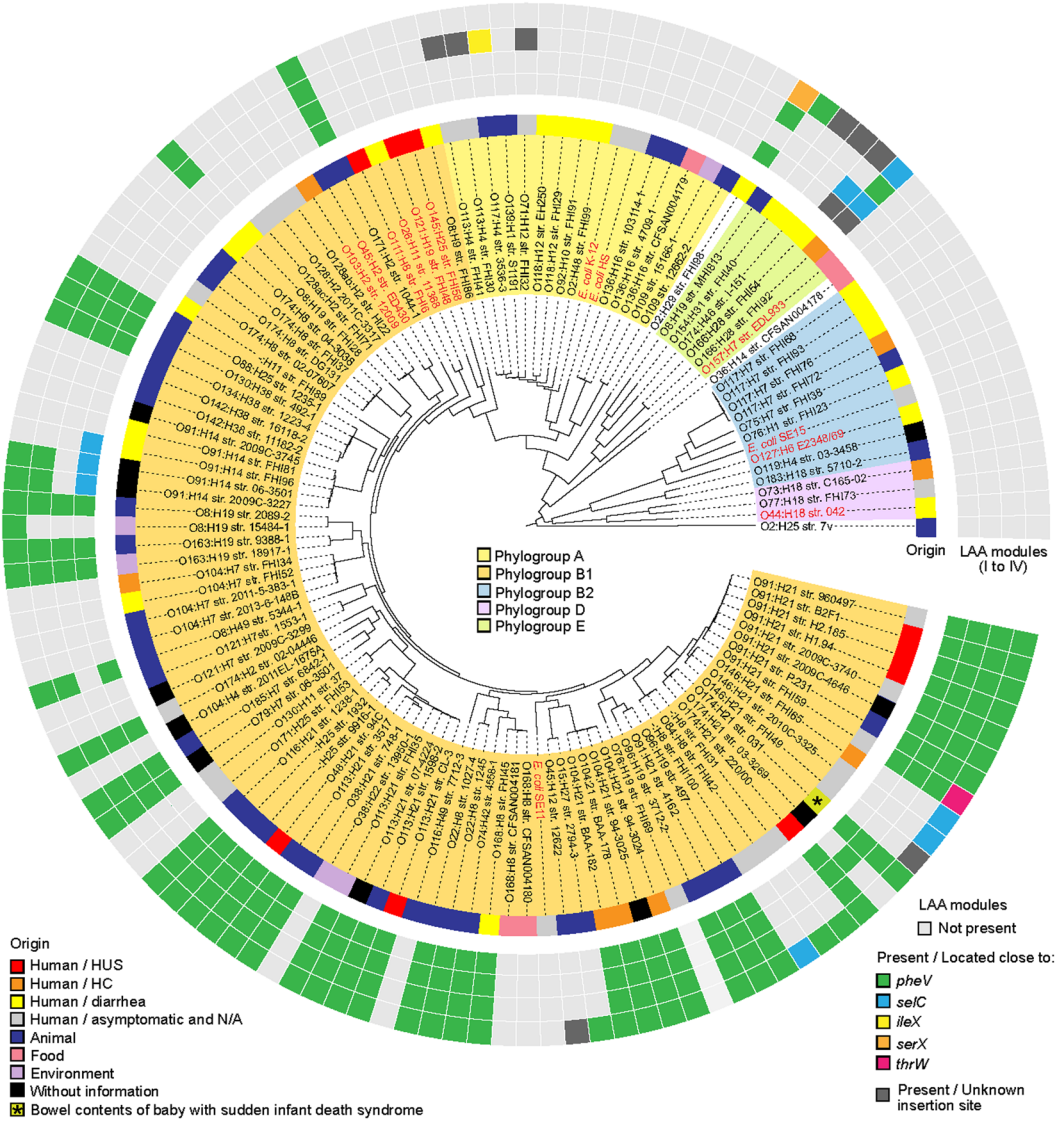
Phylogenetic relationship and distribution of LAA modules between LEE-negative STEC strains and related *E. coli* strains. A total of 128 *E. coli* genomes (115 LEE-negative STEC and 13 representative strains of other *E. coli* strains) were used to construct the maximum likelihood phylogenetic tree (midpoint rooted) based on whole genome SNPs (167,167 SNPs within 3,008,649 positions, which were found in all analyzed genomes). Phylogroups are indicated by the colors of the inner ring, according to the legend. The origin of each isolate is indicated in the middle ring by colored boxes, according to the legend. Presence of the LAA pathogenicity island or LAA modules and their insertion sites are indicated in the outermost rings by colored boxes, according to the legend. LEE-negative STEC strains and related *E. coli* strains are highlighted in black and red, respectively.

In order to evaluate the possible mobilization of LAA modules, we performed a *in silico* analysis of genome sequences (draft and complete) from 115 LEE-negative STEC strains, 7 LEE-positive STEC strains, 2 strains of other *E. coli* pathotypes, *E. coli* K-12 MG1655 and 3 commensal *E. coli* strains, which are available in the NCBI database (Supplementary Table 5) (see methods). First, all draft genome sequences were ordered and aligned using progressiveMauve. Next, phylogenetic relationships among the strains were determined based on whole-genome single nucleotide polymorphism (SNP) analysis. Additionally, phylogroup assignment was conducted *in silico* based on the methods of Clermont *et al*.^36^. Finally, a local BLASTn search was performed to determine the presence of LAA, the distribution of its modules and the tRNA loci located close to these sequences. The presence of a module was established when 50% or more of its nucleotide sequence was identified. Our results indicate that LEE-negative STEC strains are phylogenetically diverse. A maximum likelihood phylogenetic tree based on whole genome SNPs is shown in Fig. 5. In general, designation of phylogroup and serotype (antigen H) were consistent with the topology of the tree, with the exception of serotype O8:H19, in which strains were separated into different clades. In contrast, serogroups (O antigens) were polyphyletic. For example, several serotypes belonging to the serogroups O91, O113, O104 and O174 were found in distantly related clades, which is consistent with previous studies demonstrating this genetic diversity^37, 38^. Remarkably, the complete LAA structure was identified adjacent to the *pheV* gene in 34.8% (40/115) LEE-negative STEC strains from several serotypes (-: H25, O8:H19, O22:H8, O38:H21, O48:H21, O74:H42, O79:H7, O88:H25, O91:H21, O96:H19, O104:H21, O113:H21, O116:H21, O130:H38, O134:H38, O163:H19, O168:H8, O171:H2, O174:H2, O174:H21 and O185:H7), including 3 isolates from HC and 6 from HUS cases. In two additional LEE-negative STEC strains, one isolated from a HC case (O166:H28 str. FHI92) and the other from human feces (O91:H21 str. FHI59, diagnostics not available), we were also able to identify each of the four LAA modules; however they were located adjacent to different tRNA genes (*pheV, selC* and *thrW*). All the above isolates that carry the four LAA modules belong to the phylogroup B1, except the FHI92 strain, which belongs to phylogroup E. Additionally, these strains were associated (p < 0.0001) with Shiga toxin type 2 (Stx2), a toxin that is a risk factor for HUS development^8^ (Supplementary Table 6). By contrast, these strains were negatively associated (p < 0.01) with Shiga toxin type 1 (Stx1), a toxin not associated with an increased risk for HUS^8^. Interestingly, two isolates from the serotype O91:H14 lack this PAI, indicating that this locus is not present in all strains of the same serotype. Furthermore, individual LAA modules were also identified (in some cases forming part of other mosaic PAI-like structures) in several LEE-negative STEC strains, including isolates belonging to phylogroups other than B1. In these cases, the modules were also located adjacent to several tRNA genes (*pheV*, *selC*, *ileX* and *serX*), which suggest the direct acquisition and/or loss of modules. No association between these strains and a Shiga toxin type was found (Supplementary Table 6). Thus, whatever the underlying mechanism (acquisition or deletion), the analysis presented demonstrates the wide distribution and mobilization of LAA modules among LEE-negative STEC strains.

### Analysis of genetic relationships between complete LAA sequences indicates that this locus has two major lineages

**Figure 6:**
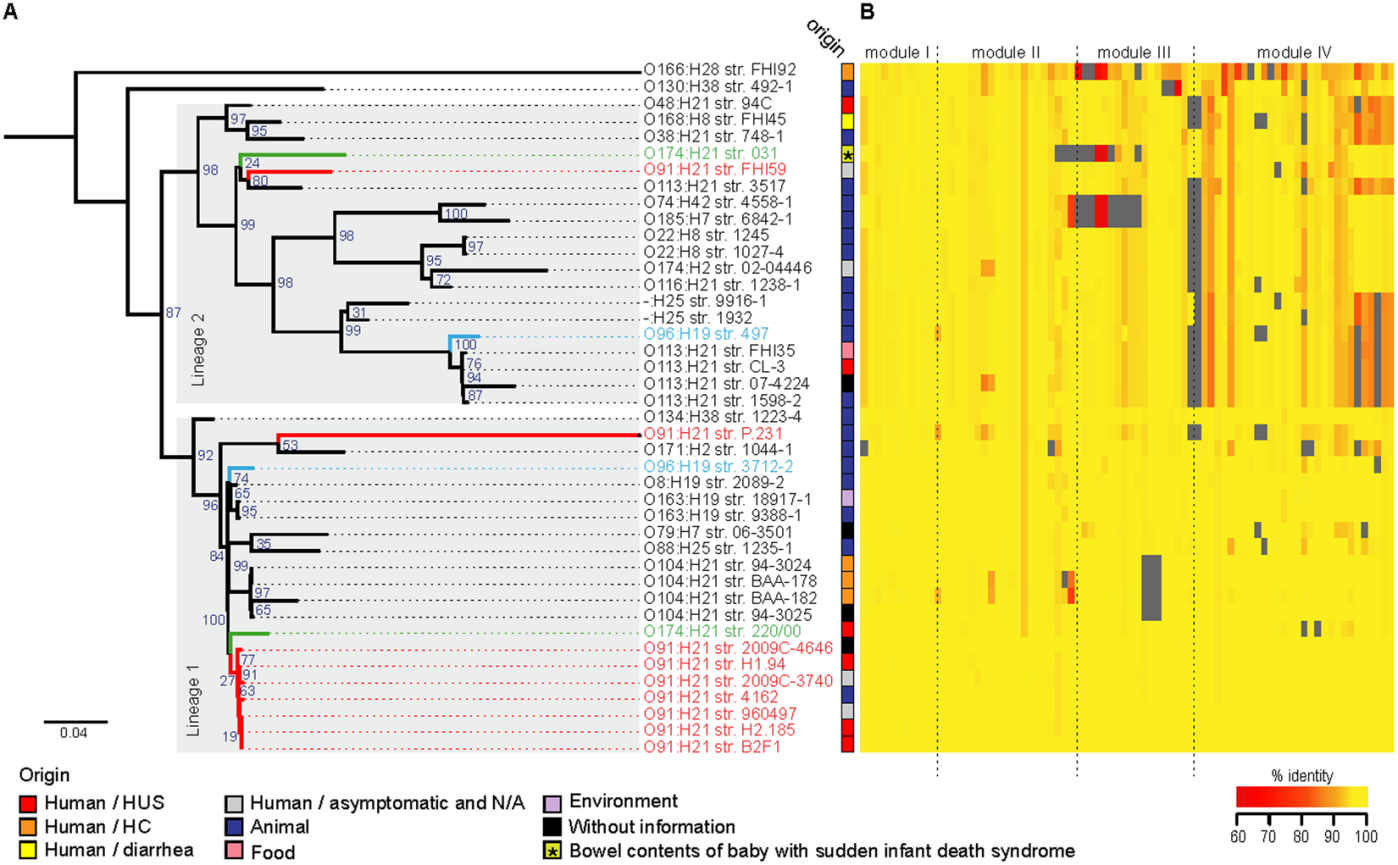
Genetic relationships of the LAA pathogenicity island and conservation of LAA-encoded genes among 42 LEE-negative STEC strains. (**A**) Maximum likelihood phylogenetic tree (midpoint rooted) of LAA sequences based on SNP analysis (677 SNPs within 49,427 positions, which were found in all sequences). The LAA_B2F1_ sequence was used as a reference. Lineages are indicated by gray shading on the branches. The origin of each isolate is indicated by colored boxes, according to the legend. Bootstrap values are indicated in the tree. Note that both lineages are present in strains belonging to serotypes O91:H21 (highlighted in red), O96:H19 (highlighted in blue) and O174:H21 (highlighted in green). (**B**) Heat map showing the presence, absence and variation of each of the 80 genes located in 42 complete LAA sequences. Each row describes the presence/absence of one isolate. Presence and variation (nucleotide identity levels, ranging from 60 to 100%) for each gene are indicated by color intensity (red to yellow), as shown in the legend. Absence was defined as an identity and/or gene coverage of less than 60%, and is indicated by the color grey. The LAA_B2F1_ sequence was used as a reference. The matrix is divided into modules, in order to show the gene variation within each module.

In order to investigate the evolutionary history of this PAI, we determined the genetic relationships between 42 LAA sequences (all four modules) based on SNP analysis. Additionally, we assessed the genetic variability of genes encoded within these sequences. In both analyses, the LAA_B2F1_ sequence was used as the reference. Notably, the phylogenetic tree of LAA sequences demonstrated that two major lineages (LAA-1 and LAA-2) are found within different strains of a same serotype, including O91:H21, O96:H19 and O174:H21 (Fig. 6A), indicating that these lineages determinations are not linked to serotype. The major genetic variability between lineages was found in modules III and IV (Fig. 6B), indicating that these DNA regions may have evolved under different evolutionary pressures. Additionally, two strains (O166:H28 str. FHI92 and O130:H38 str. 492-1) were clustered outside of the LAA-1 and LAA-2 lineages. As shown above, in the FHI92 strain, which belongs to phylogroup E, LAA modules were found adjacent to several different tRNA genes (Fig. 5). Thus, it is possible to infer that LAA modules present in the FHI92 strain may be ancestral sequences.

## Discussion

Despite persistent efforts to increase food safety, the burden of disease caused by foodborne pathogens continues to be a concern in both developed and developing countries. The evolution of these pathogens and the impact of this biological process on their epidemiology constitutes a growing challenge for public health authorities worldwide^39^. In particular, the emergence of the epidemic EAEC/STEC O104:H4 strain, which caused the 2011 outbreak in Germany, changed the epidemiology of diarrheagenic *E. coli* and showed that the genetic plasticity and horizontal gene transfer of these bacteria accelerates their adaptation to a variety of environments^40^. In this new epidemiological scenario, it is now accepted that there are LEE-negative STEC strains that represent a potential public health risk. Consequently, it is necessary to determine the molecular mechanisms by which these emerging STEC strains are causing disease in humans. Notably, our findings strongly suggest that in absence of LEE, LAA may be involved as an alternative mechanism of adhesion to the human intestine, in which Hes, Iha, Ag43 and other virulence factors encoded in this locus could participate. Owing to the biological functions of Hes (Fig. 1) and Ag43^19^, it is possible to hypothesize that LAA-mediated adherence of STEC to intestinal cells would exhibit an aggregative (or “semilocalized”) pattern, which has been reported in isolates of serotypes O91:H21 and O113:H21^41, 42^. It must be emphasized that the Hes, NmpC and Ag43 proteins, all encoded in LAA, are reactive to sera from patients with HUS^16^, indicating that they are synthetized during human disease. Thus, these antigens probably play a role in the development of these pathologies. Furthermore, the complete LAA structure was identified in strains isolated from cases of HC and HUS (Fig. 5). Besides, the first three modules of this PAI were detected through multiplex PCR assay in several LEE-negative STEC strains belonging to clinically relevant serotypes (O91:H21, O113:H21 and O174:21) and isolated from different sources, including humans, animals and foods (Supplementary Table 2). Nevertheless, because of its modular nature, LAA may be present as a “complete” (with all four modules) or an “incomplete” (with one, two or three modules) structure in different strains. Importantly, module III, previously described by Shen *et al*.^17^, has been proposed as a molecular marker of LEE-negative STEC strains linked to disease^35^. However, our data revealed that the presence of all modules, not one of them, is most probably associated with disease. It is interesting to note that the complete LAA structure was identified almost exclusively in STEC strains belong to the phylogroup B1 (Fig. 5), suggesting that a specific genetic background is required for its acquisition and/or maintenance. Furthermore, we identified two major lineages of LAA that can be present in different, but closely-related strains, including strains of a same serotype (Fig. 6A), indicating that this PAI has been most likely acquired multiple times via independent events. This supports the idea that the acquisition of LAA through horizontal gene transfer is most likely recent, which may account for the evolution and emergence of these strains. Further studies aimed at understanding the genetic variability between LAA sequences could provide more information about the evolution of this PAI and its possible role in the adaptation of *E. coli* to new niches, such as the human and bovine intestine. Accordingly, it is important to elucidate the participation (or lack thereof) of LAA in human disease, but this is beyond the scope of the present study.

To date, it has not been possible to fully define human pathogenic STEC, as there is no single or combination of marker(s), including the determination of serotype, that allows to absolutely predict the potential of a STEC strain to cause human disease^43^. However, the European Food Safety Authority Panel on Biological Hazards (BIOHAZ Panel) has suggested that the presence of molecular markers, such *stx*2 and *eae* or *aaiC* plus *aggR* along with other non-characterized markers, may be associated with a higher risk of severe disease^43^. Several virulence factors, such as *saa* ^42^, *sab* and *eibG* ^6^, and PAIs^25, 31^ have been reported as exclusively present in LEE-negative STEC strains. Nonetheless, none of these molecular markers appear to be associated with severe disease. On the contrary, our data suggest that the identification of LAA may allow the surveillance and assessment of the public health risk associated with emerging clones belonging to the group of under-diagnosed LEE-negative STEC strains. The association between LAA and Stx2 (Supplementary Table 6) is a significant result for public health considerations due to the epidemiological relevance of this toxin. This new knowledge will contribute significantly to epidemiological studies of STEC.

Moreover, the misconception that LEE-negative STEC strains make up a homogeneous subgroup has interfered with the determination of the real public health impact of these pathogens. Importantly, this study demonstrates that the current classification of STEC strains primarily based on the presence of LEE is inappropriate. Consequently, we propose a new classification scheme for STEC strains based on the presence of both the LEE and LAA pathogenicity islands, as well as the emergence of hybrid strains between STEC and other *E. coli* pathotypes^44, 45^ (Supplementary Fig. 3).

In conclusion, we demonstrate that LAA is a novel pathogenicity island that is present in a group of emerging STEC strains that cause severe diseases in humans. Our results provide evidence suggesting the involvement of LAA in the adaptation of these bacteria to the human intestine, thereby causing disease. This study contributes to an understanding of the evolution, pathogenicity and epidemiology of these human pathogens.

## Methods

### Bacterial strains, culture conditions, vectors and primers

The strains used in this study are listed in Supplementary Table 2. Strains were grown in Luria-Bertani broth (LB) or Dulbecco’s Modified Eagle’s Medium-low glucose (DMEM) at 37 °C with agitation. The culture media were supplemented as needed with ampicillin (Amp) (100 µg/mL) and/or 2 mM m-toluic acid. All vectors and primers used are listed in Supplementary Table 1. All primers designed in this study were obtained using the NCBI Primer-BLAST tool (http://www.ncbi.nlm.nih.gov).

### Detection of the *hes* gene by PCR

The presence of the *hes* gene was determined using the primers *hes_det1* + *hes_det2*, which are specific for *hes* and do not amplify other allelic variants of the Hra family (Supplementary Note 1). Positive strains for *hes* were then analyzed with the primers *hes_for* + *hes_rev* and the PCR products obtained were sequenced (Macrogen, USA).

### Cloning and expression of the *hes* and *hra1* genes

Coding sequences for *hes* and *hra1* genes were amplified using the primers *hes_clon1* + *hes_clon2* and *hra1_clon1 + hra1_clon2*, respectively. These primers allowed obtaining products with recognition sites for the restriction enzymes *Nde*I and *Bam*HI in the 5′ and 3′ ends of each gene, respectively. The *hes* and *hra1* genes were amplified from the LEE-negative O113:H21 STEC strain E045-00 and from the EAEC str. 042, respectively. PCR products were ligated to the vector pTZ57R/T (Fermentas, Lithuania), following the manufacturer’s instructions, in order to obtain the vectors pTZ57R/T_*hes* and pTZ57R/T_*hra1*. These vectors were used to transform the *E. coli* DH5α, and clones were selected according to Amp resistance and α-complementation. The correct clone was confirmed by sequencing (Macrogen, USA). Next, corresponding vectors were extracted from the transformed *E. coli* DH5α strains and digested with *Nde*I and *Bam*HI. The digestion products were analyzed by agarose gel electrophoresis, and the inserts (*hes* and *hra1* genes) were purified. These fragments were ligated to the vector pVB1 (Dualsystems Biotech, Switzerland) to obtain the vectors pVB1_*hes* and pVB1_*hra1*, in which the genes *hes* and *hra1* are regulated by the Pm/*xyl*S expression system. These vectors were used to transform the *E. coli* HB101 strain. As a control, the empty vector was also transformed in the HB101 strain.

### Functional characterization of the Hes protein

1) Hemagglutination: Agglutination of sheep erythrocytes was carried out, as previously described^46^. 2) Bacterial Autoaggregation: Bacterial autoaggregation was measured as described in ref. 21 with the following modifications. Briefly, bacterial cultures were grown in LB supplemented with the appropriate antibiotic and m-toluic acid (2 mM) overnight at 37 °C with agitation. The cultures were centrifuged at 9000 × g, re-suspended in phosphate-buffered saline (PBS) and normalized to an optical density at 600 nm (OD_600_) of ~0.9. 10 mL of each bacterial suspension was placed in two separate tubes. One tube remained static and the other was vortexed before each OD measurement. The tubes were left static at room temperature. To measure the bacterial settling over time, at designated time points (every hour for 8 hours), 0.5 mL was removed from within 1 cm of the surface of each bacterial suspension and the OD_600_ was measured. This assay was performed two times on different days. 3) Biofilm formation: Biofilm formation was observed and quantified by fixing and staining with crystal violet as described in^21^ with the following modifications. Briefly, 10 μL of overnight culture in LB supplemented with the appropriate antibiotic and m-toluic acid (2 mM) was added to 1 mL of LB medium in a 24-well plate. Plates were incubated without shaking at room temperature. At designated time points (24, 48 and 72 hours), culture medium was aspirated, each well was washed three times with PBS and contents were fixed for 7 min with 70% methanol. The wells were allowed to dry completely. Fixed biofilms were stainied with 0.5% crystal violet for 15 min and washed twice with water. Quantification of biofilm formation was carried out by the addition of 0.5 mL of 33% glacial acetic acid and measurement of the OD_595._ This assay was performed three times in triplicate on different days 4) Bacterial adhesion and invasion of human epithelial cells: Bacterial adhesion to Caco-2, HT-29 and Hep-2 cells was evaluated as previously described^46^, with slight modifications. Briefly, epithelial cells were cultivated in DMEM supplemented with 10% bovine fetal serum and 1% penicillin-streptomycin at 37 °C in 5% CO_2_ atmosphere. Cells were seeded in 24-well plates and grown to confluence (approximately 4 × 10^5^ cells/well). Bacterial pre-inoculates were grown overnight in DMEM low-glucose supplemented with the appropriate antibiotic and m-toluic acid (2 mM) at 37 °C with agitation. An aliquot of each pre-inoculum was diluted 50-fold in the same culture medium and incubated at 37 °C for 4 h with agitation. The epithelial cells were washed three times with PBS and infected with an MOI of 100 for 30 min at 37 °C in 5% CO_2_ atmosphere.

Non-adherent (planktonic) bacteria were removed by five washes with PBS, and the adherent bacteria were recovered by lysis with 0.1% Triton X-100. The number of adherent bacteria was determined by serial dilution and counts of viable bacteria in LB agar. The final result was expressed as the percentage of bacterial cells adhered to the cell layer relative to the number of bacteria added. To quantify the invasiveness of the bacterial strains, we performed a protection assay with gentamicin. One hour after infection, planktonic bacteria were removed by three washes with PBS and the cells were incubated with DMEM medium supplemented with gentamicin (50 µg/mL) for 1.5 h. The medium was removed, the cells were washed three times with PBS and the invasive bacteria were recovered by lysis with 0.1% Triton X-100. After serial dilution and a count of invasive bacteria in LB agar, we determined the percentage of invasive bacteria relative to number of bacteria added. All assays were performed three times in duplicate on different days. 5) Visualization of the adherence phenotype. HT-29 cells were cultivated on glass coverslips and infected for 1 h, as described above. After three washes with PBS, DMEM medium was added and incubated for 1 more hours. After three more washes with PBS, 70% methanol was added for 7 min and cells were stained with Giemsa 1:20 for 40 min at room temperature.

### Identification of pathogenicity islands carrying the *hes* gene

All genome sequences analyzed were downloaded from GenBank at the National Center for Biotechnology Information (NCBI - http://www.ncbi.nlm.nih.gov/) on 20 September 2016. Accession number and the source of the sequences are listed in Supplementary Table 5. Contigs of draft genomes were ordered and aligned against the complete genome of *E. coli* K-12 substr. MG1655 using progressiveMauve^33^. Then, contigs of each strain were concatenated into one contiguous sequence and the genetic context of the *hes* gene was analyzed using several bioinformatic tools. For instance, the DR sequences, IS elements and tRNA loci were identified using REPuter^47^, ISfinder^48^ and tRNAscan-SE^49^, respectively. Besides, the ORFs and the G + C content were determined by analyzing genomic sequences using Unipro GENE^50^ and the Geneious software package (v10.0.9; Biomatters Ltd). DNA with PAI features were used to performed BLASTn searches against the Pathogenicity Island Database v2.0^34^. Also, a local BLASTn search was performed in the Geneious software package to determine the distribution of LAA modules and their insertion sites in the genomes analyzed.

### Multiplex PCR assay

Simultaneous detection of the modules I, II and III of the LAA pathogenicity island was performed through PCR assay (Fig. S2). The primers LAA1_for +LAA1_rev and LAA2_for +LAA2_rev were designed to amplify modules I and II, respectively. A third pair of primers (ms3_for +ms3_rev), reported by Girardeau *et al*.^35^, were included for the amplification of module III. The amplification reactions were performed in a final volume of 25 µL containing template DNA, 0.3 µM each primer, 0.4 µM each deoxynucleoside triphosphate (Fermentas, Lithuania), 5 µL 5X GoTaq DNA polymerase buffer and 1.25 U GoTaq DNA polymerase (Promega, USA). The amplification reaction included initial denaturing at 94 °C for 5 min, 30 cycles of denaturing at 94 °C for 30 s, hybridizing at 62.5 °C for 40 s and extension at 68 °C for 2 min, with a final extension at 72 °C for 10 min. PCR products were analyzed by electrophoresis in 1% agarose gel using Tris-acetate-EDTA buffer and stained with ethidium bromide.

### SNP analysis and phylogeny

**(**1) Whole genome SNP analysis: Genome sequences, both draft and complete, were uploaded to the CSI Phylogeny 1.4 server^51^, which identifies SNPs from whole genome sequencing data, filters and validates the SNP position, and then infers phylogeny based on concatenated SNP profiles. This analysis was performed using the default input parameters and *E. coli* K-12 MG1655 as the reference genome. As a result, 167,167 SNPs were identified in 3,008,649 positions found in all analyzed genomes. The output file in Newick format was downloaded and used for visualization of the phylogenetic tree in FigTree v.1.4.2 (http://tree.bio.ed.ac.uk/software/figtree/). *In silico* PCR^52^ was performed for the determination of phylogroup based on presence/absence of the genes *chuA*, *yjaA*, *arpA*, *trpA* and the segment TspE4.C2, as proposed by Clermont *et al*.^36^. (2) Genetic relationships of the LAA pathogenicity island among LEE-negative STEC strains: A total of 42 genomic sequences of STEC strains carry the four modules of LAA were uploaded to the CSI Phylogeny 1.4 server and the SNPs identification was carried out with default input parameters and LAA_B2F1_ as the reference sequence. As a result, 677 SNPs were identified in 49,427 positions found in all sequences. Tree construction was performed as described above.

### Comparative genomic analysis

The presence, absence and variations in LAA-encoded genes was assessed by BLASTn searches performed in the Geneious software package with the LAA_B2F1_ as the reference sequence. By default, when coverage and/or identity of the genes was below to 60%, this was considered absence. Comparisons between genomes and complete LAA sequences were performed and visualized using progressiveMauve^33^ and EasyFig v2.1^53^, respectively. A heat map showing the presence, absence and identity of LAA-encoded genes was drawn using the package gplots^54^ in R^55^.

### Statistical analysis

Three independent adhesion, invasion and biofilm formation assays were performed and data was compared using the Student’s t-test (two-tailed). A *P*-value of less than 0.05 was considered significant.

## Electronic supplementary material

Supplementary Information

Supplementary Table 2

Supplementary Table 5
