# Supplementary material for: Locus of Adhesion and Autoaggregation (LAA), a pathogenicity island present in emerging Shiga Toxin–producing Escherichia coli strains
Source: Sci Rep. 2017 Aug 1;7:7011. doi: 10.1038/s41598-017-06999-y (PMC5539235; doi:10.1038/s41598-017-06999-y)
Supplement: Supplementary file 1 — Supplementary Information [file 41598_2017_6999_MOESM1_ESM.pdf]

Locus of Adhesion and Autoaggregation (LAA), a pathogenicity island present in emerging Shiga Toxin–producing *Escherichia coli* strains

David A. Montero[1], Juliana Velasco[2], Felipe del Canto[1], Jose L. Puente[3], Nora L. Padola[4], David A. Rasko[5], Mauricio Farfán[6], Juan C. Salazar[1], Roberto Vidal[1]*.

[1]Programa de Microbiología y Micología, Instituto de Ciencias Biomédicas, Facultad de Medicina, Universidad de Chile, Santiago, Chile. [2]Servicio de Urgencia Infantil, Hospital Clínico de la Universidad de Chile "Dr. José Joaquín Aguirre", Santiago, Chile. [3]Departamento de Microbiología Molecular, Instituto de Biotecnología, Universidad Nacional Autónoma de México, Cuernavaca, México. [4]Laboratorio de Inmunoquímica y Biotecnología, FCV, UNICEN, Tandil, Argentina. [5]Department of Microbiology and Immunology, University of Maryland School of Medicine, Baltimore, Maryland, USA. [6]Centro de Estudios Moleculares, Departamento de Pediatría, Hospital Dr. Luis Calvo Mackenna, Facultad de Medicina, Universidad de Chile, Santiago, Chile

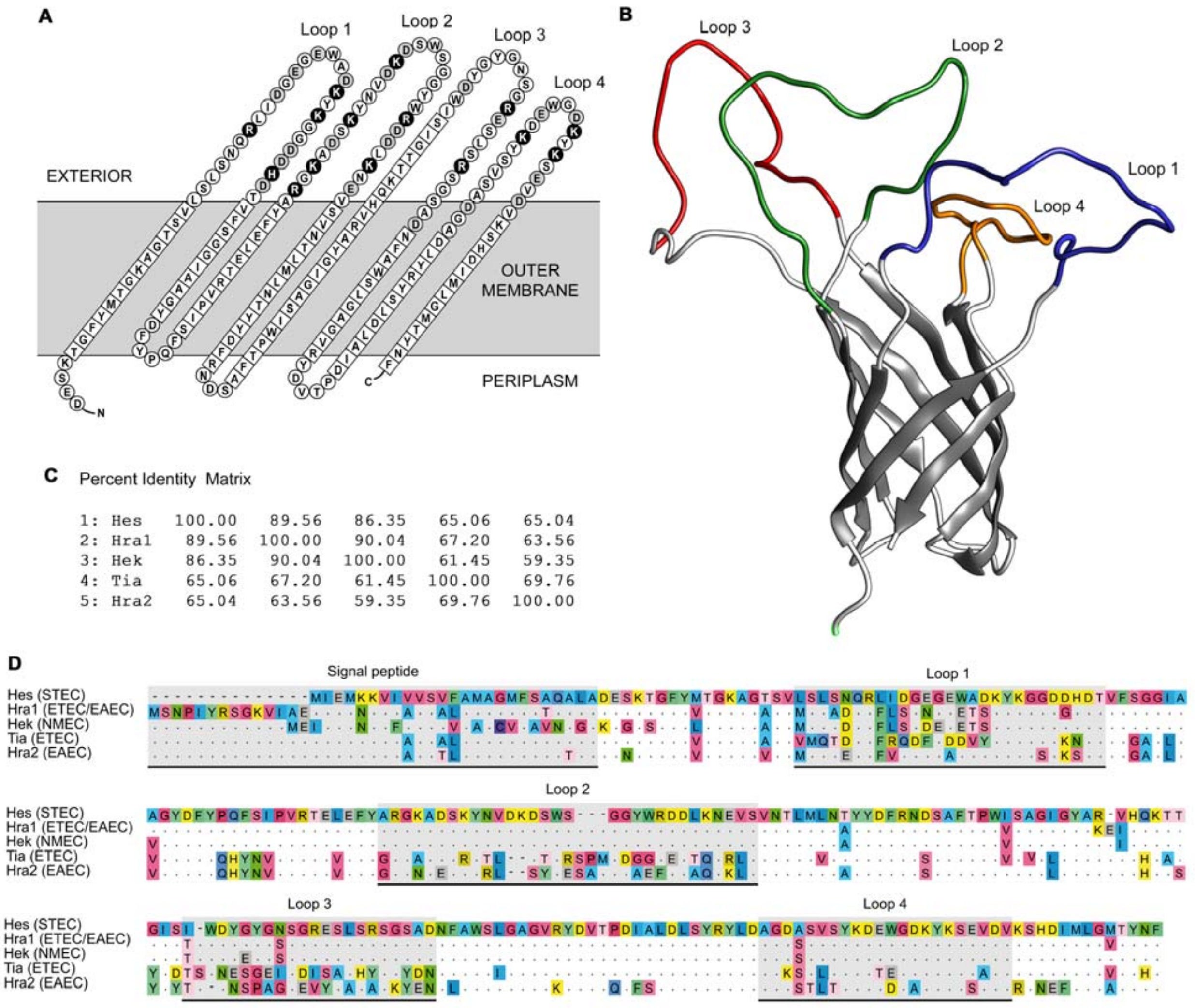

Supplementary Figure 1. The Hes protein is a new member of the Hra family. (**A**) A two-dimensional model of the topology of the mature Hes protein. Predicted amino acids that form the β-barrel transmembrane structures are indicated by squares and those that form loop structures are indicated by circles. Amino acids with positive and negative charge present in external loops (exposed to the outside) are indicated by black and gray circles, respectively. The Hes protein was modeled using Protter (http://wlab.ethz.ch/protter/start/) and PredictProtein (https://www.predictprotein.org/). The design is based on that shown for the Hek protein by Fagan et al., 2008. (**B**) Hypothetical three-dimensional structure of Hes, constructed based on the structure of the Opa60 protein (Protein Databank identification number: 2MLH) in the Phyre2 server (http://www.sbg.bio.ic.ac.uk). The image was edited using UCSF Chimera 1.10.2 (http://www.cgl.ucsf.edu/chimera). Here we show the external loops show in part (A). (**C**) Identity percentages between Hra family members. (**D**) Alignment of amino acid sequences between Hra family members using MUSCLE in Unipro UGENE 1.21 (http://ugene.net/). Residues in the alignment are colored according to the UGENE color scheme. Only residues that differ from those in the Hes sequence for the same position are shown. Points indicate identical residues Dashed indicate gaps. The accession numbers of the sequences used are Q46678 (Hra1), AAK73174 (Hek), Q46760 (Tia) and AEK70958 (Hra2).

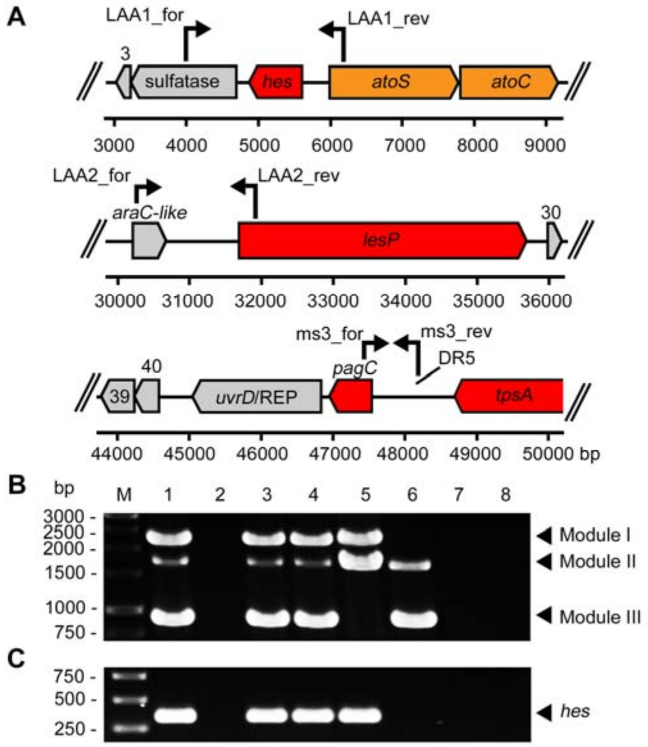

Supplementary Figure 2. Multiplex PCR detection of 3 modules of LAA. (**A**) A schematic diagram showing the regions of LAA that are amplified by multiplex PCR assays. Arrows indicate the primers used to amplify each region. Primer pairs LAA1_*for* + LAA1_*rev*, LAA2_*for* + LAA2_*rev* and ms3_*for* + ms3_*rev* were used to amplify DNA fragments located in modules I, II and III, respectively. (**B**) PCR products representative of simultaneous amplification in the first tree modules of LAA resolved by electrophoresis in agarose gel (1%). (**C**) Detection of *hes* by PCR using the primers *hes_det1* + *hes_det2*. The lines of panels b and c: 1, O113:H21 str. E045-00; 2, O113:H21 str. E045-00ΔLAA; 3, O91:H21 str. A13; 4, O104:H21 str. B45; 5, O181:H49 str. 72_1; 6, O113:H21 str. IH39595/07C; 7, O113:H21 str. 5_1; 8, negative control. M, marker of molecular size.

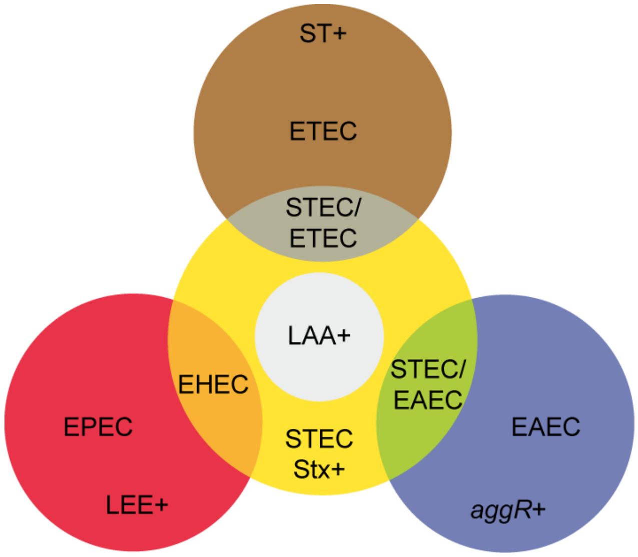

Supplementary Figure 3. Venn diagram showing relationships between STEC and other *E. coli* pathotypes. This new classification scheme for STEC is based on the presence of both LEE and LAA pathogenicity islands. Also, emergent hybrids STEC/ETEC and STEC/EAEC are shown.

Supplementary Table 1. Primers and plasmids used in this study.

Primers	Sequence 5'- 3' *	Tm (°C)	Size (pb)	Target	Reference
hes_for<br>*hes_rev*	ATGATTGAAATGAAAAAGGTTAT<br>TCAGAAGTTATAAGTCATAC	52	750	*hes*	This study
hes_det1<br>*hes_det2*	CAACCAGCGTCTTATCGAT<br>CGGTTGTTTTCTGGTGAAC	63	350	*hes*	This study
hes_clon1<br>*hes_clon2*	**CATATG**ATTGAAATAAAAAAGGTTATTGTG<br>**GGATCC**TCAGAAGTTATAAGTCATAC	53	759	*hes*	This study
hra1_clon1<br>*hra1_clon2*	**CATATG**AGCAACCCCATTTATCGTT<br>**GGATCC**TCAGAAGTTATAAGTCACAC	55	801	*hra1*	This study
LAA1_for<br>*LAA1_rev*	AACCGGGAGCCATATTCAGC<br>ACGCGGTAAGTCGATGTAGAG	63	2207	LAA Module I	This study
LAA1_for<br>*LAA1_rev*	CTGCAAAAGACATTGCCACAACA<br>TGCGACAACAGGGTCAGTGA	63	1689	LAA Module II	This study
ms-3_for<br>*ms-3_rev*	GCGTGTAGCAGCTCATGCAG<br>GACAACACTGACCGGATAATC	63	880	LAA Module III	Girardeau et al., 2009

* The underlined sequence indicates the recognition sites for the restriction enzymes *Nde*I and *BamH*I.

Girardeau JP, Bertin Y, Martin C. Genomic analysis of the PAI ICL3 locus in pathogenic LEE-negative Shiga toxin-producing *Escherichia coli* and *Citrobacter rodentium*. *Microbiology* 2009; **155**: 1016–27.

Supplementary Table 3. Open reading frames (ORFs) localized within the LAA pathogenicity island of the B2F1 strain identified by significant similarity (BLASTp search).

ORF#	Position (bp) *	Denomination in LAA PAI	Closest informative protein match	No. of identical residues / Total No. of residues (% Identity)	Accession No. of homologue	Function to closest related protein. Comments
tRNA	1-76	pheV-tRNA				
	54 - 76<br>86428 - 86450	DR1: direct repeat	TTCGATTCCGAGTCCGGGCACCA<br>TTCGATTCCGAGTCCGG-CACCA			
1	274 - 1539	*int*, integrase	Site-specific recombinase, phage integrase family	421/421 (100%)	EIH32040.1	Bacteriophage P4 integrase. Site-specific recombinase.
2	1764 - 2742	*sisA*	ShiA-like inflammation suppressor gene A	313/315 (99%)	AAN82005.1	Attenuation of host inflammatory response
3	c3013 - 3174	hypothetical protein	Inner membrane protein YhbX	50/53 (94%)	EGB65631.1	Unknown
4	c3197 - 4699	sulfatase	Arylsulfatase	483/484(99%)	EIH33024.1	Unknown
	4131 - 4146<br>36258 - 36273	DR2: Direct Repeat	AACGGTACACCCGGCA<br>AACGGAACACCCGGAA			
5	c4881 - 5630	*hes*	Heat resistant agglutinin 1	223/250 (89%)	AAC13752.1	Autoaggregation, biofilm formation, and aggregative adherence
6	6009 - 7835	*atoS*	Signal transduction histidine-protein kinase AtoS	595/608 (98%)	Q06067.1	Activates AtoC by phosphorylation
7	7832 - 9217	*atoC*	Acetoacetate metabolism regulatory protein AtoC	460/461 (99%)	Q06065.2	Involved in transcriptional regulation of ato genes for acetoacetate metabolism
8	9413 - 10075	*atoD*	Acetate CoA-transferase subunit alpha	219/220 (99%)	P76458.1	Heterodimer, involved in lipid, SCFA metabolism
9	10075 - 10725	*atoA*	Acetate CoA-transferase subunit beta	214/216 (99%)	P76459.1	Heterodimer, involved in lipid, SCFA metabolism
10	10722 - 12044	*atoE*	Short-chain fatty acids transporter	440/440 (100%)	P76460.1	Responsible for SCFA intake
11	12075 - 13259	*atoB*	Acetyl-CoA C-acetyltransferase	388/394 (98%)	P76461.1	Involved in SCFA metabolism
12	c13669 - 14025	IS66	Transposase IS66	118/118 (100%)	ERF94613.1	Transposase IS66
13	15414 - 15593	hypothetical protein	Hypothetical protein	57/57 (100%)	EGW68387.1	Unknown
14	15645 - 15818	hypothetical protein	Hypothetical protein	57/57 (100%)	EIH33102.1	Unknown
15	c16333 - 16548	hypothetical protein	Hypothetical protein	61/64 (95%)	AIF92868.1	Unknown
16	16618 - 17016	*shiD*	ColV-immunity protein	130 /132 (98%)	AAN45157.1	Colicin V immunity
	17407 - 17517	DR3: direct repeats	TTTCGTAG			
17	c17534 - 17950	*shiA*-like	ShiA-like domain protein	101/108 (94%)	EZJ33232.1	Unknown
18	18447 - 18599	hypothetical protein	Hypothetical protein	49/50 (98%)	EFW62877.1	Unknown
19	c19283 - 19465	hypothetical protein	Hypothetical protein	60/60(100%)	EGW68391.1	Unknown
20	19404 - 21491	*iha*	Bifunctional enterobactin receptor/adhesin protein	664/696 (95%)	NP_309387.1	Specific receptor of enterobactin /adhesin protein
21	c21482 - 21808	*tnpA*	Transposase TnpA	108/108 (100%)	ERF87109.1	Transposase
22	c21837 - 23681	*btuB*	TonB-dependent vitamin B12 receptor	614/614 (100%)	EGW81509.1	Vitamin B12/cobalamin outer membrane transporter
23	c24582 - 24839	hypothetical protein	Hypothetical protein	85/85 (100%)	EZA22235.1	Unknown
24	c24908 - 26026	sulfatase	Sulfatase family protein	370/372(99%)	EJK95236.1	Arylsulfatase A and related enzymes. Inorganic ion transport and metabolism
25	c26142 - 26594	arylsulfatase domain protein	Arylsulfatase domain protein	150/150(100%)	EIH33112.1	Unknown
26	c26689 - 27846	*nmpC*	Outer membrane porin protein NmpC	375/378 (99%)	ELE48963.1	Outer membrane porin protein from phage origin.
27	c28888 - 29133	hypothetical protein	Hypothetical protein	71/81 (88%)	EGW92529.1	Unknown
28	30142 - 30699	*araC*-like	AraC family transcriptional regulator	245/245 (100%)	EYZ57499.1	Transcriptional regulator
29	31598 - 35692	*lesP*	Autotransporter	1363/1364 (99%)	WP_032317126.1	Serine protease
30	35954 - 36187	hypothetical protein	Hypothetical protein	63/63 (100%)	WP_000789660.1	Unknown
31	36573 - 36764	hypothetical protein	Hypothetical protein	63/63 (100%)	WP_000264910.1	Unknown
32	36798 - 37007	hypothetical protein	Flagellar biosynthesis protein FlgM	69/69 (100%)	WP_000323309.1	Unknown
33	c37034 - 37381	IS66	Transposase, partial	115/115 (100%)	WP_000855098.1	Transposase IS66 family
34	c37505 - 38149	hypothetical protein	Hypothetical protein	214/214 (100%)	WP_001376509.1	Unknown
35	c38170 - 38439	*perC*-like	PerC transcriptional activator family protein	89/89 (100%)	KEO26191.1	Transcriptional regulator
36	c38518 - 39156	*ibrB*	Immunoglobulin-binding regulator B	212/212 (100%)	EIH32727.1	Transcriptional regulator
	39023 - 39038<br>84116 - 84131	DR4: direct repeat				
37	c39141 - 40373	*ibrA*	Immunoglobulin-binding regulator A	410/410 (100%)	EIH33651.1	Transcriptional regulator
38	c40873 - 43554	uvrD/REP helicase-like protein	Putative UvrD/REP helicase-like protein	886/893 (99%)	EKI41417.1	Superfamily I DNA and RNA helicases

39	c43805 - 44263	*impA*	Type VI secretion protein ImpA	152/152 (100%)	WP_001367976.1	Hemolysin activation/secretion protein
40	c44244 - 44621	hypothetical protein	ImpA-related N-terminal family protein, partial	115/115 (100%)	KDV76601.1	ImpA domain protein partial
41	c45046 - 46872	uvrD/REP helicase domain protein	UvrD/REP helicase domain protein	604/604 (100%)	EIH32041.1	Superfamily I DNA and RNA helicases
42	c46969 - 47544	*pagC*-like	Putative PagC-like membrane protein	182/182 (100%)	ABG02919.1	Virulence membrane protein
	48181 - 48304<br>49774 - 49887	DR5: direct repeat	TTCTTACCCTGTGCCAGAGACAGGGCTGCATTCACTACTGCATGAGCTGCTACACGCCCTGCTCCGTCCGGGGTGGTCTGTTT GATAATTTCAGCAATATACGGTGCTGCACCAGC			
43	c48729 - 57857	*tpsA*	Filamentous hemagglutinin	3042/3042 (100%)	WP_001081255.1	Hemagglutination, Adherence.
	50133 - 50148<br>57786 - 57801	DR6: direct repeat	GGCAATATCTGCCACC			
	51541 - 51556<br>52639 - 52654<br>60937 - 60952	DR7: direct repeat	TCCCTGCCGGCAATCA			
44	c57873 - 58400	*hlyC*-like	RTX toxin acyltransferase	175/175 (100%)	WP_001243916.1	RTX toxin acyltransferase family
45	c58410 - 60062	*tpsB*	Hemolysin secretion/activation protein ShlB/FhaC/HecB	550/550 (100%)	WP_001376511.1	Hemolysin activation/secretion protein
46	60738 - 60929	hypothetical protein	Z1637-like protein	63/63 (100%)	AAQ19123.1	Unknown
47	c60982 - 61215	hypothetical protein	Z1636-like protein	77/77 (100%)	AAQ19122.1	Unknown
48	c61311 - 61934	hypothetical protein	DNA-binding protein	207/207 (100%)	WP_001367955.1	DNA-binding protein
49	c62870 - 63436	hypothetical protein	Hypothetical protein	188/188 (100%)	WP_000287892.1	Unknown
50	63739 - 64041	IS600, OrfA	Transposase	100/100 (100%)	WP_000088311.1	Transposase
51	64182 - 64895	IS600, OrfB	Transposase	272/272 (100%)	WP_001367783.1	Transposase
52	c65169 - 65594	*aec59*	Hypothetical protein	141/141 (100%)	WP_001013311.1	Unknown
53	c65591 - 65974	*aec60*	Hypothetical protein	127/127 (100%)	WP_000271013.1	Unknown
54	c66235 - 66801	*aec61*	Hypothetical protein	188/188 (100%)	WP_000221486.1	Unknown
55	67005 - 67202	hypothetical protein	Hypothetical protein	65/65 (100%)	WP_000236763.1	Unknown
56	c67548 - 67688	hypothetical protein	Hemolysin activation protein, partial	46/46 (100%)	WP_000623359.1	Haemolysin expression modulating protein
57	c68195 - 68308	hypothetical protein	Hypothetical protein	37/37 (100%)	EFZ40174.1	Unknown
58	68449 - 68655	hypothetical protein	Prophage CP4-57 regulatory family protein	92/92 (100%)	EHV18472.1	Predicted DNA-binding transcriptional regulator AlpA
59	68736 - 69056	aec62, partial	Hypothetical protein, partial	104/106 (98%)	WP_033810310.1	Unknown
60	69099 - 69410	IS602, OrfA	Transposase	103/103 (100%)	WP_001036724.1	Transposase
61	69650 - 70276	IS602, OrfB	Predicted IS602 transposase OrfB	208/208 (100%)	BAI34548.1	Transposase
62	70297 - 70605	*aec62*, partial	Hypothetical protein, partial	102/102 (100%)	WP_032235550.1	Unknown
63	c70927 - 72411	*aec63*	Hypothetical protein	494/494 (100%)	WP_001297234.1	Unknown
64	72561 - 72803	hypothetical protein	Hypothetical protein	79/80 (99%)	WP_024231225.1	Unknown
65	c72843 - 72965	hypothetical protein	Hypothetical protein	40/40 (100%)	EDV62536.1	Unknown
66	73641 - 74513	*yeeP*	50S ribosome-binding GTPase family protein	290/290 (100%)	ENC40329.1	Predicted GTPase
67	74841 - 77960	*ag43*	Antigen 43	1039/1039 (100%)	EIH33235.1	Adhesion, Autoaggregation Biofilm formation
68	78081 - 80597	*aec68*	Membrane protein	838/838 (100%)	WP_001367924.1	Unknown
69	80673 - 81128	*aec69*	Hypothetical protein	148/151 (98%)	WP_000581504.1	Unknown
70	81207 - 81440	*aec70*	Hypothetical protein	77/77 (100%)	WP_001119729.1	Unknown
71	81540 - 82358	*aec71*	Hypothetical protein	271/272 (99%)	WP_001234620.1	Unknown
72	82413 - 82898	*klcA*-like	Hypothetical protein	161/161 (100%)	WP_000849590.1	Unknown
73	82899 - 83390	*yeeS*	YeeS protein	163/163 (100%)	CAE85201.1	Unknown
74	83453 - 83674	*yeeT*	Hypothetical protein	73/73 (100%)	WP_000692312.1	Unknown
75	83674 - 83787	hypothetical protein	Hypothetical protein	37/37 (100%)	WP_000488311.1	Unknown
76	83939 - 84211	*yeeU*	Antitoxin	89/90 (99%)	WP_001280952.1	Antitoxin
77	84258 - 84635	*yeeV*	Toxin	125/125 (100%)	WP_000854902.1	Toxin
78	84632 - 85120	hypothetical protein	Hypothetical protein	162/162(100%)	WP_000779175.1	Unknown
79	85132 - 85329	*aec78*	Conserved hypothetical protein	76/80 (95%)	CAX18608.1	Unknown
80	85414 - 86256	*aec79*	Restriction methylase	280/280(100%)	WP_001280537.1	Unknown

* c: indicates ORFs transcribed on the complementary strand.

Supplementary Table 4. Homologs of LesP among members of the SPATE family of autotransporters.

Homolog	Origin	Accession No.	% Identity (% Similarity)[a]			Function	Reference
			Complete Peptide	Passenger Domain	Autotransporter Domain		
EspI	STEC	CAC39286.1	76.1 (84.9)	69.6 (81.2)	96.6 (97.7)	Protease	Schmidt H, et al., 2001
EpeA	pO113	AAL18821.1	58.8 (72.7)	47.6 (65.1)	99.6 (99.6)	Mucinase	Leyton D, et al., 2003
SepA	*S. flexneri*	CAA88252.1	52.8 (67.7)	46.9 (62.2)	78.4 (91.7)	Cytotoxin	Benjelloun-Touimi Z, et al., 1995
EspP	pO157	CAA66144.1	52.1 (64.0)	40.9 (55.1)	100 (100)	Cytotoxin	Brunder W, et al.,1997
Pic	*S. flexneri* and *E. coli* O44:H18 str. 042	AAD23953.1	50.5 (66.2)	43.5 (60.2)	79.6 (90.6)	Mucinase, Serum resistant, Hemagglutination	Henderson I. et al., 1999
EatA	ETEC	AAO17297.1	50.6 (66.9)	45.9 (62.3)	73.1 (86.7)	Protease	Patel S, et al., 2004
Tsh	APEC	Q47692.1	44.4 (61.6)	40.7 (57.9)	60.2 (77.1)	Hemagglutination	Stathopoulos C, et al., 1999
TleA	ETEC	KF494347.1	43.2 (60.1)	39.0 (55.8)	60.2 (77.1)	Mucinase, Adhesion	Gutierrez G, et al., 2015

[a] Determined by EMBOSS Matcher version 2.0u4 (http://www.ebi.ac.uk/Tools/psa/emboss_matcher/). % Identity and similarity with trespect to the amino acid sequence of LesP (Accession number: EGW68403.1) present in the B2F1 strain.

Schmidt H, Zhang W-L, Hemmrich U, Jelacic S, Brunder W, Tarr PI et al. Identification and Characterization of a Novel Genomic Island Integrated at selC in Locus of Enterocyte Effacement-Negative, Shiga Toxin-Producing *Escherichia coli*. *Infect Immun* 2001; 69: 6863–6873.

Leyton DL, Sloan J, Hill RE, Doughty S, Hartland EL. Transfer Region of pO113 from Enterohemorrhagic *Escherichia coli*: Similarity with R64 and Identification of a Novel Plasmid-Encoded Autotransporter, EpeA. *Infect Immun* 2003; 71: 6307–6319.

Benjelloun-Touimi Z, Sansonetti PJ, Parsot C. SepA, the major extracellular protein of *Shigella flexneri*: autonomous secretion and involvement in tissue invasion. *Mol Microbiol* 1995; 17: 123–135.

Brunder W, Schmidt H, Karch H. EspP, a novel extracellular serine protease of enterohaemorrhagic Escherichia coli O157:H7 cleaves human coagulation factor V. *Mol Microbiol* 1997; **24**: 767–778.

Henderson IR, Czeczulin J, Eslava C, Noriega F, Nataro JP. Characterization of Pic, a secreted protease of Shigella flexneri and enteroaggregative *Escherichia coli*. Infect Immun 1999; 67: 5587–5596.

Patel SK, Dotson J, Allen KP, Fleckenstein JM. Identification and Molecular Characterization of EatA, an Autotransporter Protein of Enterotoxigenic *Escherichia coli*. *Infect Immun* 2004; **72**: 1786–1794.

Stathopoulos C, Provence DL, Curtiss R. Characterization of the avian pathogenic *Escherichia coli* hemagglutinin Tsh, a member of the immunoglobulin a protease-type family of autotransporters. *Infect Immun* 1999; **67**: 772–781.

Gutiérrez D, Pardo M, Montero D, Oñate A, Farfán M, Ruiz-Pérez F *et al.* TleA, a tsh-like autotransporter identified in a human enterotoxigenic *Escherichia coli* strain. *Infect Immun* 2015; **83**: IAI.02976-14.

Supplementary Table 6. Association between Locus of adhesion and Autoaggregation (LAA) and Shiga toxin type*

	No. strains	Shiga toxin type (No. strains)	Odds Ratio (OR)	p-value	LAA Association
LAA-positive strains (all four modules)	42	Stx1 (17)	0.33 (0.14 – 0.78)	0.009	Negative association
		Stx2 (39)	11.5 (3.13 – 42.26)	0.00001	Significant
STEC strains carrying LAA modules (<4)	24	Stx1 (16)	0.97 (0.34 – 2.73)	0.57	NS
		Stx2 (16)	1.77 (0.64 – 4.89)	0.19	NS

* LEE and LAA negative STEC strains were considered as control group (49 strains; including 33 y 26 positive strains for stx1 and stx2, respectively). Odds ratio measures how much the affected/non-affected rate increases with the factor. P-values, computed by Fisher Exact Probability Test (one-tailed), give the statistical significance of such effects. A P-value of less than 0.05 was considered significant. Last column indicates association. NS, Not significant.

Supplementary Note. As shown in the alignment of nucleotide sequences of members of the Hra family, these gene have high similarity in their 5´ and 3´ends. Therefore, in order to assess the distribution of *hes* in our culture collection, we designed the primers hes_det1 + hes_det2 (highlighted in blue), which are specific for this gene and do not amplify other allelic variants of the Hra family. Next, with the aim of evaluating the conservation of *hes*, strains positive for this gene were analyzed by PCR using the primers hes_for + hes_rev (highlighted in green), which amplify the complete nucleotide sequence of hes and other members of the Hra family, and the PCR products obtained were sequenced. The following shows the nucleotide sequences used and their alignment using Clustal Omega 1.2.4. (http://www.ebi.ac.uk/Tools/msa/clustalo/).

```
>hes, CP009106.2:c4036258-4035509 Escherichia coli strain 94-3024, complete genome
ATGATTGAAATGAAAAAGGTTATTGTGGTTTCAGTATTTGCAATGGCGGGTATGTTTTCAGCCCAGGCCT
TGGCTGATGAGAGCAAAACAGGTTTTTATATGACCGGTAAGGCGGGGACTTCCGTTTTATCTCTTTCCAA
CCAGCGTCTTATCGATGGTGAAGGAGAGTGGGCGGACAAATATAAAGGTGGTGATGACCATGATACGGTA
TTCAGTGGCGGTATCGCGGCTGGTTATGATTTTTATCCGCAGTTCAGTATTCCGGTTCGTACGGAACTGG
AGTTTTACGCTCGTGGAAAAGCTGATTCGAAGTATAACGTAGATAAAGACAGTTGGTCAGGTGGTTACTG
GCGTGATGACCTGAAGAATGAAGTGTCAGTCAACACACTGATGCTGAATACGTACTATGACTTCCGGAAT
GACAGCGCATTCACACCATGGATATCTGCAGGGATTGGCTACGCCAGAGTTCACCAGAAAACAACCGGTA
TCAGTATCTGGGATTATGGGTACGGAAACAGTGGTCGCGAATCGTTGTCACGCTCAGGCTCTGCTGATAA
CTTTGCATGGAGCCTTGGCGCAGGTGTCCGCTATGACGTCACCCCGGATATCGCGCTGGACCTCAGCTAT
CGCTATCTTGATGCAGGTGATGCCAGTGTGAGTTATAAGGACGAGTGGGGCGATAAATATAAGTCAGAAG
TTGATGTTAAAAGTCATGACATCATGCTTGGTATGACTTATAACTTCTGA

>hra1, FN554766.1:C3390633-3391424 Escherichia coli 042 complete genome
ATGAGCAACCCCATTTATCGTTCGGGGAAAGTGATAGCGGAGATGATTGAAATGAATAAGGTTATTGCGGTTTCAGCGCTTGCCATG
GCAGGCATGTTTTCGACCCAGGCTCTGGCTGATGAGAGCAAAACAGGCTTTTATGTGACCGGTAAAGCAGGTGCTTCTGTTATGTCA
CTTGCAGACCAGCGTTTCCTGTCGGGTGATGGAGAGGAAACATCAAAATATAAAGGCGGTGATGGCCATGATACGGTATTCAGTGGC
GGTATCGCGGCCGGTTATGATTTTTACCCGCAGTTCAGTATTCCGGTTCGTACGGAACTGGAGTTTTACGCTCGTGGAAAAGCTGAT
TCGAAGTATAACGTAGATAAAGACAGCTGGTCAGGCGGTTACTGGCGTGATGACCTGAAGAATGAGGTGTCAGTCAACACACTGATG
CTGAATGCGTACTATGACTTCCGGAATGACAGTGCATTCACACCATGGGTATCTGCAGGGATTGGCTACGCCAGAATTCACCAGAAA
ACAACCGGTATCAGTACCTGGGATTATGGGTACGGAAGCAGTGGTCGCGAATCGTTGTCACGTTCAGGCTCTGCTGACAACTTCGCA
TGGAGCTTTGGCGCGGGTGTCCGCTATGACGTAACCCCGGATATCGCTCTGGACCTCAGCTATCGCTATCTTGATGCAGGTGACAGC
AGTGTGAGTTACAAGGACGAGTGGGGCGATAAATATAAATCAGAAGTTGATGTTAAAAGTCATGACATCATGCTTGGTGTGACTTAT
AACTTCTGA

>hek, CP007149.1:c4848859-4848104 Escherichia coli RS218, complete genome
ATGGAGATAATTGAAATGAATAAGGTTTTTGTTGTTTCAGTGGTGGCCGCAGCCTGTGTATTTGCAGTAA
ATGCAGGAGCAAAGGAAGGTAAAAGCGGTTTTTATCTGACCGGTAAAGCCGGTGCCTCTGTGATGTCACT
TTCAGACCAGCGTTTCCTGTCAGGAGATGAGGAAGAAACATCAAAGTATAAAGGCGGCGATGACCATGAT
ACGGTATTCAGTGGCGGTATTGCGGTCGGTTATGATTTTTATCCGCAGTTCAGTATTCCGGTTCGTACAG
AACTGGAGTTTTACGCTCGTGGAAAAGCTGATTCGAAGTATAACGTAGATAAAGACAGCTGGTCAGGTGG
TTACTGGCGTGATGACCTGAAGAATGAGGTGTCAGTCAACACACTAATGCTGAATGCGTACTATGACTTC
CGGAATGACAGCGCATTCACACCATGGGTATCCGCAGGGATTGGCTACGCCAGAATTCACCAGAAAACAA
CCGGTATCAGTACCTGGGATTATGAGTACGGAAGCAGTGGTCGCGAATCGTTGTCACGTTCAGGCTCTGC
TGACAACTTCGCATGGAGCCTTGGCGCGGGTGTCCGCTATGACGTAACCCCGGATATCGCTCTGGACCTC
AGCTATCGCTATCTTGATGCAGGTGACAGCAGTGTGAGTTACAAGGACGAGTGGGGCGATAAATATAAGT
CAGAAGTTGATGTTAAAAGTCATGACATCATGCTTGGTATGACTTATAACTTCTGA

>hra2, JF808724.1:342-1088 Escherichia coli strain 60A heat-resistant agglutinin 2
(hra2) gene, complete cds; and putative regulatory protein (deoR) gene, partial cds
ATGATTGAAATGAAAAAGGTTATTGCTGTTTCAACACTCGCAATGGCAGGCATGTTTTCGGCCCAGACTC
TGGCTGATGAGAACAAAACTGGCTTTTATGTGACCGGTAAAGCCGGTGTTTCTGTTATGTCACTTTCAGA
GCAACGTTTCGTTGATGGTGAAGGGGCATGGGCGGATAAATACAAAGGCAGTGACAAAAGTGATACGGTT
TTTGGCGCAGGCCTTGCGGTGGGATACGATTTTTATCAGCACTACAATGTGCCGGTGCGTACTGAAGTGG
AGTTTTATGGCCGCGGTAACGCTGAATCAAAATATCGTCTGAGTTATTGGGAAAGTGCCGGTGGTGCCGA
GTTCGATGATGCACAGAACAAGCTCAGTGTAAACACGCTGATGCTGAATGCGTATTATGATTTCAGAAAC
AGCAGTGCATTCACGCCATGGATATCTGCTGGTCTGGGTTATGCACGGGTTCATCATAAAACTTCGTACA
TATATACCGATAACAGCCCGGCAGGCAGTGAAGTTTATTCAGCTTCAGCGTCAAAATACGAAAACAACCT
GGCATGGAGCCTGGGTGCCGGTGTTAAATACGATGTGACGCAGGATTTCAGCCTTGACCTCAGCTACCGG
TATCTGGATGCAGGGGATTCCACTCTGACTTATAAAGATGAGGATGGTGCTAAATATAAATCCTCTGTTG
```

```
ATGTCAGAAGTAACGAGTTTATGTTAGGCGCAACGTATAACTTCTGA

>tia, U20318.1 Escherichia coli tia invasion determinant gene, complete cds
ATGATTGAAATGAAAAAGGTTATTGCGGTTTCAGCGCTTGCAATGGCAGGTATGTTTTCGGCCCAGGCTC
TGGCTGATGAGAGCAAAACAGGCTTTTATGTGACCGGTAAAGCCGGTGCTTCAGTTGTGATGCAGACTGA
CCAGCGCTTCCGTCAGGACTTTGGGGATGATGTTTATAAGTATAAGGGCGGTGATAAAAACGATACTGTA
TTTGGTGCCGGCCTTGCAGTGGGCTATGATTTTTATCAACATTACAATGTTCCAGTACGCACGGAAGTGG
AATTCTATGGCCGTGGAGCTGCAGACTCCCGTTATACACTGGATACATGGCGTTCTCCGATGGGGGATGG
TGGTCGGGAAGACACACAAAATAGGCTCAGTGTGAATACCCTGATGGTGAACACGTATTATGATTTCAGA
AACAGCAGTGCATTTACTCCATGGGTATCTGTTGGCCTGGGTTATGCACGGGTACATCATAAGGCGACAT
ATATTGATACCTCCTGGAATGAATCTGGCGAGATAAGTGATATTTCTGCGTTACATTACTCGGGATATGA
TAACAACTTCGCATGGAGCATTGGGGCCGGTGTTCGCTATGACGTAACCCCGGATATCGCTCTTGACCTC
AGCTATCGCTATCTGGATGCTGGTAAATCCTCCCTGTCTTACAAGGATACAGAAGGGGATAAATATAAAT
CAGAGGCTGACGTTAAAAGTCATGACATTATGCTTGGGGTAACTTATCATTTCTGA



CLUSTAL O(1.2.4) multiple sequence alignment

hes       ------------------------------------------ATGATTGAAATGAAAAAG
hra1      ATGAGCAACCCCATTTATCGTTCGGGGAAAGTGATAGCGGAGATGATTGAAATGAATAAG
hek       ------------------------------------ATGGAGATAATTGAAATGAATAAG
hra2      ------------------------------------------ATGATTGAAATGAAAAAG
tia       ------------------------------------------ATGATTGAAATGAAAAAG
                                                    ** *********** ***

hes       GTTATTGTGGTTTCAGTATTTGCAATGGCGGGTATGTTTTCAGCCCAGGCCTTGGCTGAT
hra1      GTTATTGCGGTTTCAGCGCTTGCCATGGCAGGCATGTTTTCGACCCAGGCTCTGGCTGAT
hek       GTTTTTGTTGTTTCAGTGGTGGCCGCAGCCTGTGTATTTGCAGTAAATGCAGGAGCAAAG
hra2      GTTATTGCTGTTTCAACACTCGCAATGGCAGGCATGTTTTCGGCCCAGACTCTGGCTGAT
tia       GTTATTGCGGTTTCAGCGCTTGCAATGGCAGGTATGTTTTCGGCCCAGGCTCTGGCTGAT
          *** ***  ******     * **    **  *  * *** *     *  *    **  *

hes       GAGAGCAAAACAGGTTTTTATATGACCGGTAAGGCGGGGACTTCCGTTTTATCTCTTTCC
hra1      GAGAGCAAAACAGGCTTTTATGTGACCGGTAAAGCAGGTGCTTCTGTTATGTCACTTGCA
hek       GAAGGTAAAAGCGGTTTTTATCTGACCGGTAAAGCCGGTGCCTCTGTGATGTCACTTTCA
hra2      GAGAACAAAACTGGCTTTTATGTGACCGGTAAAGCCGGTGTTTCTGTTATGTCACTTTCA
tia       GAGAGCAAAACAGGCTTTTATGTGACCGGTAAAGCCGGTGCTTCAGTTGTGATGCAGACT
          **    ****  ** ****** ********** ** **    ** **  *    *   *

hes       AACCAGCGTCTTATCGATGGTGAAGGAGAGTGGGCGGACAAATATAAAGGTGGTGATGAC
hra1      GACCAGCGTTTCCTGTCGGGTGATGGAGAGGAAACATCAAAATATAAAGGCGGTGATGGC
hek       GACCAGCGTTTCCTGTCAGGAGATGAGGAAGAAACATCAAAGTATAAAGGCGGCGATGAC
hra2      GAGCAACGTTTCGTTGATGGTGAAGGGGCATGGGCGGATAAATACAAAGGCAGTGACAAA
tia       GACCAGCGCTTCCGTCAGGACTTTGGGGATGATGTTTATAAGTATAAGGGCGGTGATAAA
           * ** **  *       *     *  *           ** ** ** **  * **

hes       CATGATACGGTATTCAGTGGCGGTATCGCGGCTGGTTATGATTTTTATCCGCAGTTCAGT
hra1      CATGATACGGTATTCAGTGGCGGTATCGCGGCCGGTTATGATTTTTACCCGCAGTTCAGT
hek       CATGATACGGTATTCAGTGGCGGTATTGCGGTCGGTTATGATTTTTATCCGCAGTTCAGT
hra2      AGTGATACGGTTTTTGGCGCAGGCCTTGCGGTGGGATACGATTTTTATCAGCACTACAAT
tia       AACGATACTGTATTTGGTGCCGGCCTTGCAGTGGGCTATGATTTTTATCAACATTACAAT
             ***** ** **  * *  **  * ** *  ** ** ******** *  ** * ** *

hes       ATTCCGGTTCGTACGGAACTGGAGTTTTACGCTCGTGGAAAAGCTGATTCGAAGTATAAC
hra1      ATTCCGGTTCGTACGGAACTGGAGTTTTACGCTCGTGGAAAAGCTGATTCGAAGTATAAC
hek       ATTCCGGTTCGTACAGAACTGGAGTTTTACGCTCGTGGAAAAGCTGATTCGAAGTATAAC
hra2      GTGCCGGTGCGTACTGAAGTGGAGTTTTATGGCCGCGGTAACGCTGAATCAAAATATCGT
tia       GTTCCAGTACGCACGGAAGTGGAATTCTATGGCCGTGGAGCTGCAGACTCCCGTTATACA
           * ** ** ** ** *** **** ** ** *  ** **    ** ** **    ***

hes       GTAGATAAAGACAGTTGGTCA---GGTGGTTACTGGCGTGATGACCTGAAGAATGAAGTG
hra1      GTAGATAAAGACAGCTGGTCA---GGCGGTTACTGGCGTGATGACCTGAAGAATGAGGTG
hek       GTAGATAAAGACAGCTGGTCA---GGTGGTTACTGGCGTGATGACCTGAAGAATGAGGTG
hra2      CTGAGTTATTGGGAAAGTGCC---GGTGGTGCCGAGTTCGATGATGCACAGAACAAGCTC
tia       CTGGATACATGGCGTTCTCCGATGGGGGATGGTGGTCGGGAAGACACACAAAATAGGCTC
```

```
            *   *             *    ** * *         ** **     * **     *

hes        TCAGTCAACACACTGATGCTGAATACGTACTATGACTTCCGGAATGACAGCGCATTCACA
hra1       TCAGTCAACACACTGATGCTGAATGCGTACTATGACTTCCGGAATGACAGTGCATTCACA
hek        TCAGTCAACACACTAATGCTGAATGCGTACTATGACTTCCGGAATGACAGCGCATTCACA
hra2       AGTGTAAACACGCTGATGCTGAATGCGTATTATGATTTCAGAAACAGCAGTGCATTCACG
tia        AGTGTGAATACCCTGATGGTGAACACGTATTATGATTTCAGAAACAGCAGTGCATTTACT
              ** ** ** ** *** ****  **** ***** *** * **   *** ***** **

hes        CCATGGATATCTGCAGGGATTGGCTACGCCAGAGTTCACCAGAAAACAACCGGTATCAGT
hra1       CCATGGGTATCTGCAGGGATTGGCTACGCCAGAATTCACCAGAAAACAACCGGTATCAGT
hek        CCATGGGTATCCGCAGGGATTGGCTACGCCAGAATTCACCAGAAAACAACCGGTATCAGT
hra2       CCATGGATATCTGCTGGTCTGGGTTATGCACGGGTTCATCATAAAACTTCGTACATATAT
tia        CCATGGGTATCTGTTGGCCTGGGTTATGCACGGGTACATCATAAGGCGACATATATTGAT
           ****** **** *  **  * ** ** **  *  * ** ** **  *  *    **   *

hes        ATCTGGGATTATGGGTACG---GAAACAGTGGTCGCGAATCGTTGTCACGCTCAGGCTCT
hra1       ACCTGGGATTATGGGTACG---GAAGCAGTGGTCGCGAATCGTTGTCACGTTCAGGCTCT
hek        ACCTGGGATTATGAGTACG---GAAGCAGTGGTCGCGAATCGTTGTCACGTTCAGGCTCT
hra2       ACCGATAACAGCCCGGCAGG------CAGTGAAGTTTATTCAGCTTCAGCGTCAAAATAC
tia        ACCTCCTGGAATGAATCTGGCGAGATAAGTGATATTTCTGCGTTACATTACTCGGGATAT
           * *               *        ****         *          **    *

hes        GCTGATAACTTTGCATGGAGCCTTGGCGCAGGTGTCCGCTATGACGTCACCCCGGATATC
hra1       GCTGACAACTTCGCATGGAGCTTTGGCGCGGGTGTCCGCTATGACGTAACCCCGGATATC
hek        GCTGACAACTTCGCATGGAGCCTTGGCGCGGGTGTCCGCTATGACGTAACCCCGGATATC
hra2       GAAAACAACCTGGCATGGAGCCTGGGTGCCGGTGTTAAATACGATGTGACGCAGGATTTC
tia        GATAACAACTTCGCATGGAGCATTGGGGCCGGTGTTCGCTATGACGTAACCCCGGATATC
           *   * *** * ********* * ** ** *****    ** ** ** ** * **** **

hes        GCGCTGGACCTCAGCTATCGCTATCTTGATGCAGGTGATGCCAGTGTGAGTTATAAGGAC
hra1       GCTCTGGACCTCAGCTATCGCTATCTTGATGCAGGTGACAGCAGTGTGAGTTACAAGGAC
hek        GCTCTGGACCTCAGCTATCGCTATCTTGATGCAGGTGACAGCAGTGTGAGTTACAAGGAC
hra2       AGCCTTGACCTCAGCTACCGGTATCTGGATGCAGGGGATTCCACTCTGACTTATAAAGAT
tia        GCTCTTGACCTCAGCTATCGCTATCTGGATGCTGGTAAATCCTCCCTGTCTTACAAGGAT
              ** *********** ** ***** ***** **  *   *    **   ***  ** **

hes        GAGTGGGGCGATAAATATAAGTCAGAAGTTGATGTTAAAAGTCATGACATCATGCTTGGT
hra1       GAGTGGGGCGATAAATATAAATCAGAAGTTGATGTTAAAAGTCATGACATCATGCTTGGT
hek        GAGTGGGGCGATAAATATAAGTCAGAAGTTGATGTTAAAAGTCATGACATCATGCTTGGT
hra2       GAGGATGGTGCTAAATATAAATCCTCTGTTGATGTCAGAAGTAACGAGTTTATGTTAGGC
tia        ACAGAAGGGGATAAATATAAATCAGAGGCTGACGTTAAAAGTCATGACATTATGCTTGGG
                 ** * ********* **    * *** ** * **** * **  * *** * **

hes        ATGACTTATAACTTCTGA
hra1       GTGACTTATAACTTCTGA
hek        ATGACTTATAACTTCTGA
hra2       GCAACGTATAACTTCTGA
tia        GTAACTTATCATTTCTGA
              ** *** * ******
```
